# B-cell targeted therapeutics in clinical development

**DOI:** 10.1186/ar3906

**Published:** 2013-04-04

**Authors:** Stephan Blüml, Kathleen McKeever, Rachel Ettinger, Josef Smolen, Ronald Herbst

**Affiliations:** 1 Department of Rheumatology, Medical University of Vienna, Vienna, Austria; 2 2nd Department of Medicine, Hietzing Hospital, Vienna, Austria; 3 MedImmune, LLC, Department of Research, One MedImmune Way, Gaithersburg, MD 20854, USA

## Abstract

B lymphocytes are the source of humoral immunity and are thus a critical component of the adaptive immune system. However, B cells can also be pathogenic and the origin of disease. Deregulated B-cell function has been implicated in several autoimmune diseases, including systemic lupus erythematosus, rheumatoid arthritis, and multiple sclerosis. B cells contribute to pathological immune responses through the secretion of cytokines, costimulation of T cells, antigen presentation, and the production of autoantibodies. DNA-and RNA-containing immune complexes can also induce the production of type I interferons, which further promotes the inflammatory response. B-cell depletion with the CD20 antibody rituximab has provided clinical proof of concept that targeting B cells and the humoral response can result in significant benefit to patients. Consequently, the interest in B-cell targeted therapies has greatly increased in recent years and a number of new biologics exploiting various mechanisms are now in clinical development. This review provides an overview on current developments in the area of B-cell targeted therapies by describing molecules and subpopulations that currently offer themselves as therapeutic targets, the different strategies to target B cells currently under investigation as well as an update on the status of novel therapeutics in clinical development. Emerging data from clinical trials are providing critical insight regarding the role of B cells and autoantibodies in various autoimmune conditions and will guide the development of more efficacious therapeutics and better patient selection.

## Introduction

**Figure 1:**
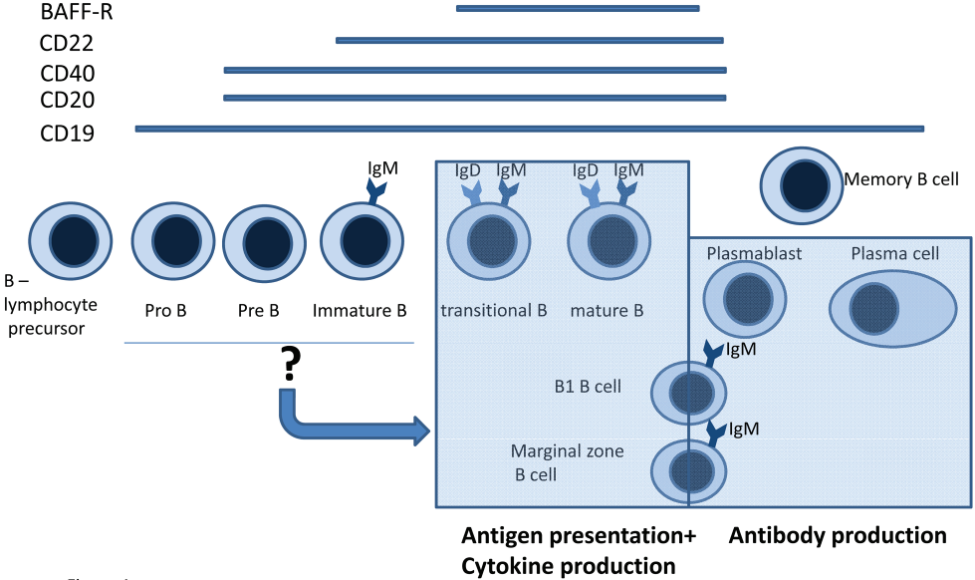
**Schematic representation of B-cell differentiation and maturation states**. Schematic representation of B-cell differentiation and maturation states with respect to expression of CD19 and CD20, CD22, CD40 and B-cell activating factor receptor (BAFF-R) as well as their functions as discussed in the main text. There is of course a variety of additional surface markers characterizing various subpopulations of B cells (for reviews see [4,12]).

B cells play a central role in the adaptive immune response and protection against pathogens. However, it is now evident that B cells also contribute to the pathobiology of many autoimmune diseases. B cells are not a homogeneous population of lymphocytes, but rather are a mixture of cells at different stages of maturation along the lineage (Figure 1) and with unique functional properties. In healthy individuals, B-cell homeostasis and the representation of different B-cell subsets in peripheral blood and lymphoid organs is finely balanced. In autoimmune diseases, however, B-cell homeostasis and activation state can be significantly altered and self-tolerance lost.

The demonstration that B-cell depletion with the CD20 antibody rituximab can lead to significant benefit to patients with rheumatoid arthritis (RA) has provided the original proof of concept for the targeting of B cells in autoimmune diseases. Although we still do not yet fully understand all aspects of B-cell contribution to disease and the mechanisms that can lead to the loss of B-cell tolerance, the pioneering studies with rituximab have led to a great variety of new approaches to target B cells with mAbs and other biologics, and many of these new molecules are currently undergoing testing in the clinic.

The following sections provide an overview of the current status of B-cell targeting biologics in the clinic. Importantly, one has to appreciate the large variety of B-cell subpopulations in the course of B-cell differentiation, activation, regulation, and function, as well as respectively characteristic molecules. This is particularly pertinent for the understanding and interpretation of data from clinical trials in different autoimmune diseases. While one can make various assumptions on the importance of certain targets from the physiological perspective and/or information obtained from studies in experimental models, it is the results of clinical trials that will provide the ultimate evidence for or against the efficacy and safety of a specific targeted therapy and, consequently, also insight into the true pathogenetic involvement of the respective pathway.

B cells can contribute to autoimmune disease through a variety of different mechanisms, including autoantibody production, antigen presentation, and cytokine production. Therapies focusing on B cells may thus have a variety and varying effects depending on the molecule or sub population targeted. To this end, it is essential to briefly highlight the rationale of these therapies in light of the diversity of the function of B cells and their subpopulations as well as addressing consequences of such therapeutics that may be of a more general nature and not necessarily related to a specific target.

B cells are the unique cell family capable of producing immunoglobulins (Figure 1). Once activated by antigens via the B-cell receptor (BCR), B cells also express other immunoglobulin isotypes as BCRs, dependent on their respective commitment. Immunoglobulin secretion then becomes a quality of plasma cells (PCs), but B1 and MZ B cells can also secrete IgM (Figure 1). Immunoglobulins are a central element in host defense. However, many autoimmune diseases are characterized by the production of autoantibodies that are either directly responsible for cell or organ damage or are characteristic for certain autoimmune diseases without (as yet) sufficiently understood pathogenic roles. This nature renders these diseases susceptible to B-cell targeted therapies in practice or theory.

PCs are only a small fraction of the total B lymphocyte pool (about 1%). However, they are responsible for the production of almost all immunoglobulins [1]. PCs are thought to arise mainly in response to T-cell-dependent antigens, although the existence of PCs after B-cell activation by T-cell-independent antigens has been reported [2]. As PCs migrate to the bone marrow, they become terminally differentiated, gain CD138 expression, and express low or no HLA. These cells are believed to be capable of living in the bone marrow for decades and providing humoral immunity to antigens seen over a lifetime.

Importantly, the frequency of plasmablasts/PCs in peripheral blood has been linked to the response of patients to B-cell targeted therapies in several studies (see below), and the various therapeutics in development may differ with regard to their impact on PCs and auto-antibody levels.

Some controversy exists regarding the existence of yet another antibody-producing B-cell population related to MZ B lymphocytes - B1 B cells, which at least in mice are a major subset [3,4]. B1 B cells, which can be subdivided into CD5^+ ^B1a and CD5^- ^B1b cells (B1 cells not expressing CD5), mainly respond to antigens and produce antibodies that are independent of T-cell help (TI antigens, as opposed to T-dependent antigens) [5]. These B1 B cells deliver so-called natural antibodies that are frequently self-reactive, and therefore have been implicated in the secretion of various autoantibodies characteristic of autoimmune diseases [5-9]. While the majority of data concerning B1 B cells have been obtained in mice, a number of autoimmune phenomena in humans, including the production of autoantibodies such as rheumatoid factor, have been associated with CD5^+ ^B cells. However, the overall role of B1 B cells in human immunology and contribution to disease is still a matter of debate [8-10]. B2 B cells (also termed follicular B cells) are the classical B cells and can be found in all secondary lymphoid organs and in the blood [3].

Antigen presentation is a fundamental activity in the generation of the adaptive immune response [11,12]. Antigens can be presented by a variety of cell populations, be it cells that become antigen-presenting cells as a secondary or bystander effect or professional antigen-presenting cells, such as B lymphocytes.

To execute their primary function in the immune response - the production of high-affinity antibodies - B cells need the help of antigen-specific T cells, which is MHC class II restricted and involves multiple co-stimulatory signals such as CD40/CD40 ligand (CD40L) and ICOS/ICOS ligand [11-13]. The main route for antigen acquisition by B cells is via the BCR. This BCR signaling can be enhanced by antigens that have cleaved C3 complement attached to them via binding to CD21 and co-recruitment of CD19 [14], or can be inhibited by the inhibitory receptor CD32B (Fcγ receptor (FcγR) IIb) [15]. Secondly, B cells can acquire antigen via immunoglobulin-independent mechanisms such as pinocytosis [16].

The interaction of B cells with MHC class II restricted CD4^+ ^antigen-specific T-helper cells renders them capable of class switch recombination and affinity maturation. These processes activate B cells, leading to upregulation of co-stimulatory molecules such as CD80 and CD86. Consequently, B cells become fully equipped antigen-presenting cells with the capacity to activate T cells [17-19].

B cells appear to have an essential role for antigen presentation in the context of antibody generation, but not for primary T-cell activation. Furthermore, in murine models of human RA, B cells have been shown to be involved in the generation of (auto)antigen-specific T-cell responses and were necessary for priming of arthritogenic T cells [20-22].

B cells are also a source of cytokines that shape the immune response. After activation, B cells produce proinflammatory cytokines such as IL-1, IL-6, granuloctye-macrophage cerebrospinal fluid and TNF, but also immunosuppressive ones such as transforming growth factor beta and, importantly, IL-10 [23,24]. Studies in human and mice suggest that B-cell-derived cytokines are able to shape polarization of T cells. B-cell-derived IL-12 has been shown to augment IFNγ production by human T cells. Of note, this effect was independent of BCR crosslinking, but dependent on T-cell-derived CD40L and microbial stimuli (activators of Toll-like receptor 9), suggesting that antigen-unspecific bystander activation can influence T-cell polarization [25].

Lymphotoxin and TNF expressed by B cells are required for the maintenance of lymphoid follicles in Peyers patches of adult mice [26,27]. B cells are also a source of receptor activator of NF-κB ligand, an essential regulator of osteoclastogenesis [28]. Increased osteoclastogenesis leading to local bone destruction is an important pathogenic aspect in multiple myeloma, but also in inflammatory arthritis [29-31].

Apart from these proinflammatory and immunostimulating roles of B-cell-derived cytokines, abundant data exist on the role of B cells in inhibiting or dampening an immune response. B cells that inhibit various (immune) pathologies have been termed regulatory B cells (Bregs) in analogy to regulatory T cells (see article by Kalampokis and colleagues, this issue) [32-34]. The most important mediator of the effects of Bregs is IL-10, but transforming growth factor beta might also be involved [35,36]. There is thus ample evidence that the role of B cells in immunity as well as autoimmunity is not confined to the production of (auto)antibodies, but that this cell type plays important roles in shaping the outcome of physiological as well as pathological immune responses (Figure 1). Therapeutic approaches currently pursued in the clinic are not yet geared towards targeting specific B-cell subsets, but have nonetheless already generated some very promising results in a variety of autoimmune indications.

## Specific therapeutic approaches to target B cells

### B-cell depletion

#### Mechanisms of antibody-mediated B-cell depletion: antibody-dependent cellular cytotoxicity and complementdependent cytotoxicity

The depleting activity of rituximab, and several other B-cell-targeted mAbs (see below), largely rely on two mAb Fc-dependent mechanisms: antibody-dependent cellular cytotoxicity (ADCC) and complement-dependent cytotoxicity (CDC). The engagement of effector cells by therapeutic mAbs requires the interaction of the mAb Fc with FcγRs on the surface of natural killer cells, monocytes/macrophages, or neutrophils. The most relevant activating FcγRs in human are FcγRIIA and FcγRIIIA [37,38]. Importantly, there is a clear relationship between polymorphisms in FcγRIIA and FcγRIIIA and the clinical response to rituximab-based therapy [39,40]. Consistent with the findings in patients with hematologic malignancies, B-cell depletion in systemic lupus erythematosus (SLE) patients homozygous for the low-affinity allele was much less efficient as compared with heterozygous patients or patients homozygous for the high-affinity allotype. B-cell depletion in homozygous F158/F158 patients, however, did improve with increasing doses of rituximab [41]. These findings further emphasize ADCC as an important factor for mAb activity, providing the rationale to apply protein engineering technology to generate new molecules with significantly improved potency.

The ADCC activity of therapeutic mAbs can be enhanced by increasing the affinity of the mAb Fc for activating FcγRs, in particular FcγRIIIA [42]. This can be achieved by the introduction of point mutations in the Fc or by modification of the Fc carbohydrate, specifically eliminating the fucose moiety [37,43,44]. Both approaches significantly increase the affinity of human IgG_1_ to the low-affinity allotype of FcγRIIIA and thus have the potential to further improve the efficacy of therapeutic mAbs [45-47]. ADCC enhancement technology has now been applied to a variety of mAbs, and several of the engineered molecules are at various stages of clinical development (see below).

In addition to ADCC, CDC is another mechanism by which therapeutic mAbs can mediate target cell killing. Activation of the classical complement pathway requires binding of the complement component C1q to the mAb Fc. Opsonization of the target cell then results in recruitment of additional complement components and formation of the membrane attack complex, which generates a pore in the plasma membrane, leading to nonapoptotic cell death [48].

#### B-cell depletion by targeting CD20 with rituximab

**Figure 2:**
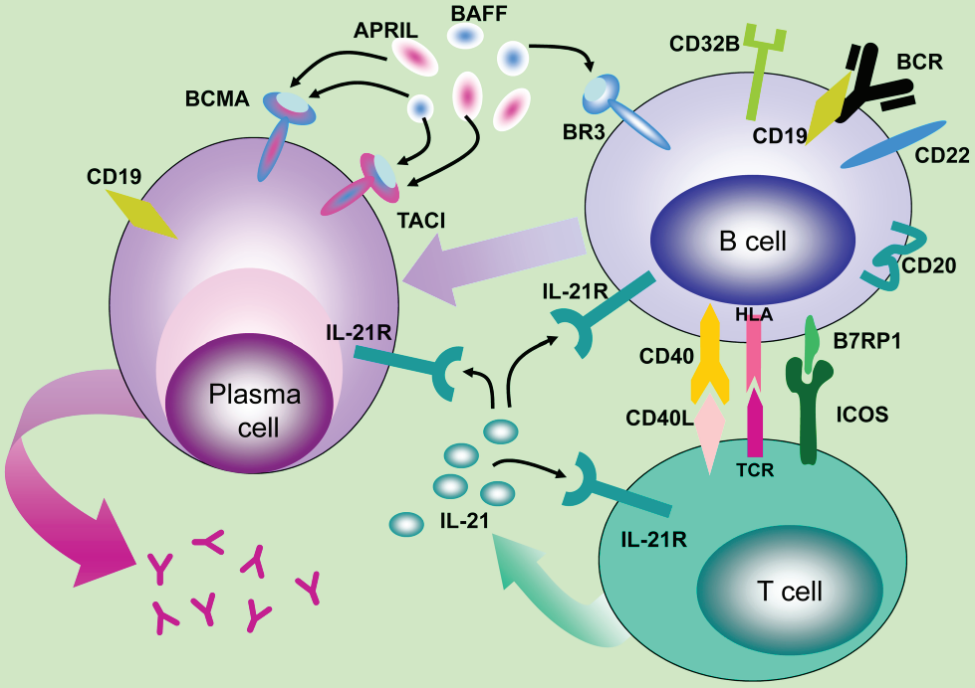
**B-cell antigens and cytokines targeted by biologics in clinical development**. Schematic representation of B-cell/T-cell interaction and differentiation of activated B cells into antibody secreting plasma cells. B cells presenting antigen to T cells via HLA receive co-stimulatory signals from T-cell-expressed CD40 ligand (CD40L). CD4 T cells, in particular T follicular helper (T_FH_) cells, in turn receive activating signals from the B-cell-expressed ICOS ligand B7RP-1. Class switch recombination by B cells and plasma cell differentiation are critically dependent on IL-21 and co-stimulation through the CD40/CD40L pathway. The two TNF family members B-cell activating factor (BAFF) and a proliferation-inducing ligand (APRIL) provide survival signals by triggering their respective receptors expressed on B cells (BAFF receptor BR3 and transmembrane activator and calcium-modulating ligand interactor (TACI) on memory B cells) and plasma cells (BAFF/APRIL receptors B-cell maturation (BCMA) and TACI). B cells can also be directly targeted by antibodies against B-cell restricted antigens, such as CD20, CD22, and CD19. See text for additional details. BCR, B-cell receptor; TCR, T-cell receptor.

Rituximab, a mouse/human chimeric IgG_1_ mAb, was the first B-cell targeting therapeutic antibody approved by the US Food and Drug Administration. Originally developed for the treatment of B-cell malignancies [49,50], rituximab demonstrated clinical activity in RA, leading to its subsequent approval in moderate to severe RA with inadequate response to TNF antagonists. In 2011 rituximab was also approved for the treatment of anti-neutrophil cytoplasmic antibody-associated vasculitides, such as granulomatosis with polyangiitis (Wegener's syndrome) and microscopic polyangiitis [51,52]. Rituximab targets the B-cell-restricted surface antigen CD20 and leads to rapid and profound B-cell depletion (Figure 2) [53,54]. Much of what we have learned about B-cell depletion over the past decade is based on preclinical and clinical data generated with rituximab.

CD20 is expressed on the majority of B cells in circulation and lymphoid tissues, including immature, mature, and memory B cells (Figure 1). CD20, however, is not expressed on lymphoid progenitors in the bone marrow, which allows for repopulation of B cells following rituximab therapy. The repopulation of B cells following rituximab therapy has been investigated in the context of several clinical studies and has provided significant insight into the ontogeny of human B-cell development [55-58]. In RA patients treated with rituximab, newly emerging B cells are of an immature and naïve phenotype [55,59]. Similar observations have been made in patients with Sjögren's syndrome, SLE, and B-cell lymphoma [56,57,60]. Interestingly, at repopulation the transitional B cells dominate and are increased in number over normal or pretreatment levels. Furthermore, the results consistently show a delayed recovery of CD27^+ ^memory B cells, with numbers below normal for up to 2 years following treatment with rituximab. Roll and colleagues also reported the recirculation of CD20^- ^PCs in parallel with the accumulation of transitional cells in the periphery [59].

Importantly, the results from these and other studies also provide critical insight into how the clinical response to rituximab treatment may be linked to the pattern of B-cell depletion and repopulation. Leandro and colleagues observed that RA patients experiencing disease relapse at the time of B-cell repopulation had a higher frequency of blood memory B cells as compared with patients without relapse [55]. Similar observations were made by Roll and colleagues, who also noted that a high frequency of memory B cells at baseline is associated with earlier relapse [59]. Consistent with this observation, another RA study showed a better clinical response to rituximab in patients with low CD27^+ ^memory B-cell numbers at baseline [61]. Also in patients with SLE the presence of memory cells as well as plasmablasts at the time of repopulation after rituximab therapy was associated with early relapse [62]. Collectively, the available data emphasize that the efficiency of depletion - in particular, of memory B cells from blood and lymphoid organs - is an important factor determining the quality of response to rituximab-mediated B-cell depletion.

B-cell depletion with rituximab is relatively well tolerated, even though some patients have sustained blood B-cell depletion for more than 6 months. As with any immunosuppressive therapy, an increased risk for severe or opportunistic infections is a major concern. Surprisingly, the overall risk for severe infections appears to be comparable with the placebo population in several clinical trials, although in some studies the risk of serious infection was about 50% higher than in the placebo group [63,64]. The analysis of data from 1,303 patients in the French Autoimmunity and Rituximab (AIR) registry, which comprises patients in clinical practice rather than clinical trials, however, identified low serum IgG levels at baseline as a risk factor for the development of severe infections following rituximab therapy [65].

Patients can also develop secondary hypogammaglobulinemia during the course of repeat cycles of rituximab treatment, which may also increase infection risk in these patients [63]. Hypogammaglobulinemia is perhaps caused by the inability to generate new memory B cells and PCs, which probably leads to a depletion of the pool of short-lived PCs over time. In addition to bacterial infections, very rare cases of progressive multifocal leukoencephalopathy (PML) have been reported with rituximab use [63,66,67]. PML is a demyelinating infection caused by reactivation of the endemic John Cunningham virus and is fatal in many cases. PML is most often seen in patients that are severely immunosuppressed, either as a consequence of disease (AIDS) or strong immunosuppressive drugs. Apart from rituximab, PML has also been observed in autoimmune patients treated with anti-TNF, natalizumab, disease-modifying anti-rheumatic drugs, and alkylating agents [68]. While rare, PML is a devastating disease and thus requires continued vigilance and awareness. Case reports have suggested that rapidly instituted plasma pheresis to eliminate mAb in natalizumab-associated PML can lead to recovery from the disease [69,70].

Another potential safety concern of B-cell depletion with rituximab is an impairment in vaccine responses, which has been explored in several recent studies. The available data clearly demonstrate that rituximab impairs the humoral response to influenza and other vaccines. Further, it appears that the impact is dependent on the level of B-cell depletion or repopulation at the time of immunization [71-73]. Pre-existing antibody titers were not affected, however, again reflecting the lack of depletion of CD20^- ^PCs with rituximab. Prophylactic vaccination against pathogens should thus be considered before initiation of B-cell depletion therapy. Important to keep in mind is that potential safety concerns with rituximab probably also apply to other therapeutics that broadly deplete B cells (see below).

In addition to RA, numerous small clinical studies have also demonstrated benefit of rituximab in patients with SLE [74-81]. Similarly, a prospective analysis of patient data from the French AutoImmunity and Rituximab (AIR) registry demonstrated clinical efficacy in SLE [82]. Promising results were also obtained in patients with lupus nephritis [82-86]. Two large randomized controlled clinical trials, one in SLE (the EXPLORER trial) and one in lupus nephritis (the LUNAR trial), not reaching their primary endpoints therefore came as a surprise [87-89]. The outcome of these trials stimulated intense discussion in the field. The use of B-cell-depleting mAbs in lupus nephritis and in the EXPLORER and LUNAR trials is discussed in great detail in the article by Reddy and colleagues (this issue). In brief, several factors may have influenced the outcome of these trials, and the same factors may also influence the success of other therapeutics currently in clinical development for the treatment of SLE. One factor is clearly the heterogeneity of the disease with its many different manifestations (see American College of Rheumatology (ACR) criteria for diagnosis of SLE) and varying organ involvement. Better patient selection (inclusion or exclusion of certain organ involvements) with consideration of response to prior immuno suppressive therapy and autoantibody as well as complement status will be important in future trials.

One should also note that, in the context of clinical trials, B-cell depletion is typically only monitored in peripheral blood. We therefore do not know to what extent B cells were depleted in secondary lymphoid organs. Complete B-cell depletion in blood and tissues may, however, be required to achieve the best possible clinical response. Another factor may be the inability of rituximab to deplete autoantibody-producing CD20^- ^CD19^+ ^PCs (see also sections 'Targeting CD19 for B-cell depletion' and 'Effects beyond B cells').

Another major factor is also the use of active therapy, such as corticosteroids, in the placebo arm, which may mask, at least in part, the activity of the investigational drug. For example, the LUNAR trial explored the activity of rituximab plus mycophenolate (cellcept) versus mycophenolate alone. A drug that interferes with lymphocyte function, mycophenolate does have a profound effect on B-cell activation and PC differentiation, which would make it difficult to see effects of B-cell targeting agents such as rituximab [90]. Another important issue is the available tools for outcome measurements. The standard disease activity indices, such as those of the SLE Disease Activity Index and the British Isles Lupus Assessment Group (BILAG), were not designed for the measurement of improvements as a response to therapy in clinical trials and are thus far from optimal. The SLE Response Index used in the belimumab trials (see below) is also based on these disease activity instruments. Of major concern, therefore, is the development of new and improved tools for the measurement of SLE disease activity and response to treatment. Patient management and drug development in SLE could also be facilitated by validated biomarkers to monitor specific organ involvement and drug response. While significant progress has been made in this area, candidate biomarkers still need validation in prospective clinical studies [91].

Rituximab did generate promising data in several small open-label and controlled trials in primary Sjögren's syndrome, an autoimmune disease characterized by inflammation of the exocrine glands. The salivary and lacrimal glands are the ones most affected, but Sjögren's syndrome patients often also present with extraglandular manifestations and multiorgan involvement. Sjögren's syndrome is further characterized by several autoantibodies - such as anti-SSA (anti-Ro), anti-SSB (anti-La) and rheumatoid factor - and B-cell hyperactivity [92]. B cells as well as T cells are present in the affected salivary glands and are often found in aggregates or ectopic lymphoid structures. In addition, the presence of PCs, which may produce autoantibodies locally, in the diseased tissue has been demonstrated. The early studies with rituximab demonstrated that B-cell depletion can improve salivary gland function, as well as extraglandular symptoms, including fatigue and arthralgia [93]. Improvement in salivary flow, however, was dependent on residual gland function. Unfortunately, these encouraging results could not be confirmed in a randomized and placebo-controlled trial (the TEARS study), the results of which were recently reported at the Annual European Congress of Rheumatology (EULAR) [94]. In contrast to the rituximab trials in SLE, the placebo arm in the TEARS study did not include active therapy. More research is clearly needed to better understand the underlying pathobiology and the potential for B-cell targeted therapies in primary Sjögren's syndrome.

Rituximab has also been evaluated in multiple sclerosis (MS) with promising results. While rituximab is not being pursued further in this indication, the data provided the rationale for testing other CD20 mAbs in MS. The MS data obtained with rituximab are briefly summarized in the section on ocrelizumab (see below).

#### Ongoing clinical studies with rituximab

Rituximab is currently being evaluated in several additional autoimmune indications, with phase 3 trials ongoing in pemphigus (ClinicalTrials.gov:NCT00784589), autoimmune hemolytic anemia (AIHA) (ClinicalTrials.gov:NCT01181154), and immune thrombocytopenia (ITP) (ClinicalTrials.gov:NCT00344149) (Table 1).

**Table 1:** B-cell targeting biologics in clinical development

**Target **	**Therapeutic **	**Format **	**Mechanism **	**Indication **	**Clinical stagea^a^**
CD20	Rituximab	Chimeric	Depletion	RA	Approved
		IgG_1_	ADCC, CDC	Pemphigus	Phase 3
				AIHA	Phase 3
				ITP	Phase 3 (newly diagnosed)
				ITP	Phase 2
				Acute IPF	Phase 1/2 (with plasma exchange)
				AIR	Phase 1
	Ofatumumab	Humanized	Depletion	RA	Phase 3
		IgG_1_	ADCC, CDC	RRMS	Phase 2
				RRMS	Phase 2 (subcutaneously)
	Ocrelizumab	Humanized	Depletion	RRMS	Phase 3
		IgG_1_	ADCC, CDC	PPMS	Phase 3
				SLE	Phase 3
	Veltuzumab	Humanized	Depletion	RA	Phase 2
		IgG_1_	ADCC, CDC	ITP	Phase 1/2
CD19	MEDI-551	Humanized	Depletion	SSc	Phase 1
		IgG_1 _afucosylated	ADCC	RRMS	Phase 1
CD22	Epratuzumab	Humanized	Partial depletion	SLE	Phase 3
		IgG_1_	B-cell activation		
BAFF	Belimumab	Human	Ligand neutral	SLE	Approved
		IgG_1_	Survival	SLE	Phase 3 (subcutaneously)
				ITP	Phase 2
				MG	Phase 2
	Tabalumab	Human	Ligand neutral	SLE	Phase 3 (subcutaneously)
IgG4			Survival	RA	Phase 2
	Blisibimod	Peptibody	Ligand neutral	SLE	Phase 3
				ITP	Phase 2/3
BAFF/APRIL	Atacicept	Receptor	Ligand neutral	SLE	Phase 2/3
		Fc fusion	Survival		
CD40L	CDP7657	Pegylated	Co-stimulation	SLE	Phase 1
		Fab	Blockade		
B7RP1	AMG-557	Human	Co-stimulation	SCLE	Phase 1
		IgG	Blockade	Psoriasis	Phase 1
				SLE	Phase 1
ICOS	MEDI-570	Human	Depletion	SLE	Phase 1
		IgG_1 _afucosylated	ADCC		
IL-21	NN8828	Humanized	Ligand neutral	RA	Phase 1
		IgG_1_			

ADCC, antibody-dependent cellular cytotoxicity; AIHA, autoimmune hemolytic anemia; AIR, autoimmune retinopathy; APRIL, a proliferation-inducing ligand; BAFF, B-cell activating factor; CD40L, CD40 ligand; CDC, complement-dependent cytotoxicity; IPF, idiopathic pulmonary fibrosis; ITP, idiopathic thrombocytopenia purpura; MG, myasthenia gravis; PPMS, primary progressive multiple sclerosis; RA, rheumatoid arthritis; RRMS, relapsing remitting multiple sclerosis; SLE, systemic lupus erythematosus; SSc, systemic sclerosis; SSLE, subacute cutaneous lupus erythematosus. ^a^Source for clinical trial status: www.clinicaltrials.gov (as of February 2013).

Pemphigus is a rare autoimmune blistering (or bullous) disease with several subtypes and is characterized by the presence of specific autoantibodies [95]. The two main subtypes are pemphigus vulgaris, which can be fatal and where sores often occur in the mouth, and pemphigus foliaceus, characterized by crusty skin sores. In all forms of pemphigus, autoantibodies form against desmogleins (Dsgs), which belong to the cadherin superfamily of adhesion molecules. Dsgs are components of desmosomes, which form intercellular junctions and are import ant for the structural integrity of tissues [96]. In pemphigus foliaceus, autoantibodies form against Dsg1, which is expressed in the epidermis; while pemphigus vulgaris is characterized by autoantibodies against Dsg3, present in skin and mucous membranes, and in some subtypes also by anti-Dsg1 [97,98].

Several small uncontrolled trials have evaluated rituximab in patients with pemphigus vulgaris and pemphigus foliaceus, and the published results on 103 patients with pemphigus vulgaris and 20 patients with pemphigus foliaceus have recently been summarized by Schmidt and colleagues [99]. The overall results are quite encouraging and most patients experienced a clinical benefit from rituximab-mediated B-cell depletion. Healing of all lesions was observed in 77% of pemphigus vulgaris patients and 40% of patients were eventually taken off immunosuppressive drugs [99]. Encouraging were also the responses in pemphigus foliaceus, where skin lesions healed completely in 85% of patients treated with rituximab [99]. Rituximab did not have a significant effect on total serum IgG but did lower IgM. More importantly, complete responses were associated with reductions in anti-Dsg1 autoantibodies, while anti-Dsg1 titers did not change or increase in patients relapsing after rituximab treatment [100]. Results from the ongoing phase 3 trial will hopefully confirm these early data and demonstrate the benefit of B-cell depletion in pemphigus.

AIHA and ITP (formerly idiopathic thrombocytopenia purpura) are two conditions in which blood cells are destroyed by autoantibodies. AIHA is a relatively rare condition in which the patient develops autoantibodies against red blood cells, leading to their destruction via complement activation or phagocytes of the reticuloendothelial system [101]. Similarly, autoantibodies against surface antigens, most commonly against the fibrinogen receptor glycoprotein IIb/IIIa, lead to the destruction of platelets in ITP [102,103].

Given the low incidence of AIHA, only limited data from small patient cohorts are available for the use of rituximab in this indication [104]. Collectively, however, the results from these studies are encouraging, with reported overall response rates ranging from 40 to 100%. Rituximab also appeared effective in a more recent Belgian retrospective multicenter study that included 53 AIHA patients, consistent with previous reports [105]. Together, the available data provide a strong rationale for the ongoing phase 3 clinical trial.

As for AIHA, corticosteroids are the standard initial treatment for patients with ITP. For ITP patients, intravenous immunoglobulin or anti-D therapy is also used to maintain safe platelet counts. For many years, splenectomy has been the main option for patients who are refractory to or relapsing from first-line therapy [106]. While splenectomy is highly effective, more and more patients appear to seek alternative options. Auger and colleagues recently conducted a meta-analysis of the results from 19 retrospective or prospective observational studies of rituximab in nonsplenectomized ITP patients [107]. Overall, rituximab appeared to be well tolerated and effective, with an overall response rate of 57%, complete response of 41%, and median duration of response of 49 weeks. Rituximab does not appear to be curative, but relapsing patients generally respond well to retreatment with the CD20 mAb [108]. Interestingly, a fairly rapid response has been observed in some patients following rituximab administration, with platelet counts rebounding within <4 weeks [107]. This has led Stasi and colleagues to hypothesize that in early responders B cells opsonized with rituximab block the macrophage system, allowing platelets to recover during the mAb infusion phase [109]. The reduction of autoantibodies as a consequence of B-cell depletion would then account for the late response to treatment. Why the opsonization effect and early response is seen in only some patients and not in all patients is currently unknown [109]. The available data indicate that B-cell depletion therapy may be a viable option to delay (or avoid) splenectomy for ITP and AIHA patients.

#### B-cell depletion with the CD20 mAb ocrelizumab

Ocrelizumab (rhumAb 2H7v.16) is a humanized CD20 mAb, which binds a different but overlapping epitope from rituximab. Ocrelizumab has similar CDC but two fold to fivefold increased ADCC compared with rituximab [110]. Ocrelizumab appears to elicit relatively fewer anti-drug antibody responses as compared with the chimeric CD20 mAb rituximab [111,112]. This second-generation CD20 mAb has been tested in patients with RA.

In a first randomized placebo-controlled phase 1/2 trial (ClinicalTrials.gov:NCT00077870) in patients receiving concomitant methotrexate, ocrelizumab was well tolerated and effective [111]. Clinical responses according to the ACR criteria were noted for all doses. The highest response rate of 50% ACR20 at week 24, relative to 22% in the placebo group, was observed with the highest doses of 500 mg and 1,000 mg. All doses resulted in rapid and profound B-cell depletion in peripheral blood, but the re-emergence of B cells was dose dependent up to a dose of 200 mg. At doses ≥200 mg, no differences were observed with regard to the kinetics of B-cell recovery. Ocrelizumab treatment resulted in a minor reduction of serum IgM levels but had no obvious effect on IgA and IgG. Doses ≥200 mg also resulted in a notable reduction of serum C-reactive protein levels [111].

Following these encouraging data, several phase 3 RA trials have been initiated: two in patients with inadequate response to methotrexate (STAGE, ClinicalTrials.gov: NCT00406419; FEATURE, ClinicalTrials.gov:NCT 00673920), one testing ocrelizumab in conjunction with methotrexate in methotrexate-naïve patients (FILM, ClinicalTrials.gov:NCT00485589), and one testing ocrelizumab in patients with an inadequate response to at least one TNF inhibitor (SCRIPT, ClinicalTrials.gov: NCT 00- 4 76996) [113-115]. The STAGE trial achieved its primary and secondary endpoints of ACR20 improvement and reduction in joint damage, respectively. However, an increased rate of serious infections relative to placebo was observed in these trials [113-115]. Given the unfavorable benefit-risk profile, the development of ocrelizumab in RA has been terminated. A phase 3 study of ocrelizumab in lupus nephritis patients, the BELONG study (ClinicalTrials.gov:NCT00626197), is still ongoing. No safety or efficacy data from this trial have yet been published.

Although safety concerns have halted the development of ocrelizumab in RA, clinical trials in relapsing remitting multiple sclerosis (RRMS) and the primary progressive form of MS are ongoing. The development of ocrelizumab in this indication builds on the promising data that have been generated earlier with rituximab and have generated significant excitement in the field [116,117] (Table 1). MS is an inflammatory demyelinating disease of the central nervous system and can lead to progressive and long-term disability [118]. In most patients the disease begins with a relapsing course with complete or partial recovery (remission). Patients eventually enter a progressive phase with continuous degradation (secondary progressive MS), but in about 15% of patients the disease is of progressive nature from the beginning (primary progressive MS) [118]. While the cause of the disease is unknown, inflammation is prominent - especially in RRMS. While earlier concepts attributed a major role to T cells in disease pathogenesis, it has become evident that B cells and humoral responses also play an important role in MS [116,117,119-121].

Importantly, the results obtained with rituximab further support the notion that B cells do play a critical role in RRMS. In the HERMES trial, rituximab significantly reduced total gadolinium-enhancing lesions at all time points for primary analysis (weeks 12, 16, 20, and 24) and the reduction was sustained for 48 weeks. In addition, the frequency of relapses was significantly improved with rituximab relative to placebo [122]. Interestingly, reductions in inflammatory lesions were observed as early as 4 weeks after treatment. While peripheral B cells were completely depleted, serum immunoglobulin levels were not affected [122]. How longer term depletion with anti-CD20 affects antibody titers and patient outcome in RRMS remains to be seen. In the OLYMPUS trial that tested rituximab in primary progressive MS, however, the results were less clear cut [123]. The primary endpoint, time to disease progression over a 96-week treatment period, was not met. Subgroup analysis, however, demonstrated a significant effect of rituximab in younger patients (<51 years of age), especially those with more prominent inflammation based on gadolinium-enhancing lesions [123].

The results from a phase 2, randomized placebo-controlled trial of ocrelizumab in patients with RRMS were recently published [124]. This trial compared two doses of ocrelizumab, a total dose of 600 mg and of 2,000 mg given in two infusions on days 1 and 15, versus IFNβ1a, given once per week by intramuscular injection, and placebo. In ocrelizumab-treated patients, the number of lesions was lower by 89% and 96% for the 600 mg and 2,000 mg groups, respectively [124]. Both doses of ocrelizumab were also better in reducing gadolinium-enhancing lesions than FNβ1a. The frequency of serious infections was similar for all cohorts in the trial, which did not use background immunosuppressive drugs as was the case in the RA trials. A caveat of this study is the lack of dose response with regard to the reduction of gadolinium-enhancing lesions (see also ofatumumab in RRMS in the following section). Based on this parameter, the activity of ocrelizumab appeared to be maximal at the lower 600 mg dose, so it is still unclear what the optimal dose might be.

Currently, two phase 3 trials in RRMS (ClinicalTrials. gov:NCT01247324, ClinicalTrials.gov:NCT01412333) and one phase 3 trial in primary progressive MS (Clinical-Trials.gov:NCT01194570) are ongoing, all of these with 600 mg ocrelizumab given in two infusions 2 weeks apart. Another question is the effect of ocrelizumab on disability parameters, which was not addressed in phase 2 trials but has been incorporated in the phase 3 studies. This will be an important variable in the evaluation of clinical benefit beyond reduction of central nervous system in flammation and brain lesions. The longer trials with larger patient number will also address the infection risk in this patient population.

#### Depletion with the CD20 mAb ofatumumab

Ofatumumab (2F8, HuMax-CD20) is a fully humanized IgG_1 _mAb, which binds an epitope on CD20 that is distinct from the rituximab binding site [125]. Binding of this unique epitope appears to influence the functional characteristics and is probably responsible for the enhanced CDC activity of ofatumumab [126-128]. Preclinical studies demonstrated that ofatumumab can mediate CDC with rituximab-resistant cell lines and with cells expressing low levels of CD20 [127]. Ofatumumab has been evaluated in several clinical trials in non- Hodgkin's lymphoma and chronic lymphocytic leukemia, where the mAb demonstrated safety and activity, leading to its approval by the US Food and Drug Administration for the treatment of chronic lymphocytic leukemia in 2009.

In a phase 1/2 study of ofatumumab in RA patients with an inadequate response to disease-modifying antirheumatic drugs, the mAb appeared safe and effective [129]. All treated groups had a significantly higher ACR20 response compared with placebo, but there was no clear dose response for ACR criteria between the 300 mg, 700 mg and 1000 mg doses tested. B cells were depleted within 1 week with depletion lasting >24 weeks. B-cell depletion did not result in changes of serum IgG and IgA, but a minor reduction in serum IgM was noted [129]. In a follow-up phase 3 study (ClinicalTrials. gov:NCT00291928), 700 mg ofatumumab was tested in biologic-naïve RA patients with an inadequate response to methotrexate [130]. By week 24 the ACR20 response was 50% versus 27% in the placebo group. No unexpected safety findings were observed in this study [130] (ClinicalTrials.gov:NCT00655824). A separate phase 3 trial is investigating the efficacy of ofatumumab in RA patients who had an inadequate response to anti-TNF therapy (ClinicalTrials.gov:NCT00603525) (Table 1).

A phase 2 dose-finding study of ofatumumab in RRMS has been completed (OMS115102, ClinicalTrials.gov: NCT01526993). The trial evaluated 100 mg, 300 mg and 700 mg mAb administered by intravenous infusion. Despite the low patient numbers in each cohort, ofatumu mab showed a substantial reduction in new as well as total lesions relative to placebo at week 24 [131].

The lack of a clear dose response, also noted above for ocrelizumab, is probably a result of the dose range tested, the number of patients per cohort, together with the increased potency of ocrelizumab and ofatumumab over rituximab. All dose levels of ofatumumab tested in the phase 2 RRMS study resulted in complete depletion of B cells from peripheral blood, although a dose-dependent recovery was noted, with more blood B cells detectable in the 100 mg dose cohort at week 24 as compared with the higher dose levels [131]. The dose cohorts in the ofatumumab phase 2 trial were small (7 to 11 patients per arm), however, and a dose-dependent effect could have been more obvious with greater sample size and/or with lower doses. In the case of ocrelizumab, doses ≥200 mg (given twice 2 weeks apart) in the RA phase 2 trial had similar kinetics of B-cell depletion and recovery, indicating that depletion of B cells from the blood and secondary lymphoid organs is already maximal at the 200 mg dose [111]. The kinetics of B-cell depletion and recovery could be different in MS patients, but depletion data were not discussed in detail in the report by Kappos and colleagues [124]. Also, neither study reported the impact on B cells in the central nervous system, such as in the cerebrospinal fluid, or effects on autoantibodies or oligoclonal bands within the cerebrospinal fluid, which could have provided further insight with regard to mechanism and efficacy. For ofatumumab a second phase 2 study (the MIRROR study) testing a subcutaneous formulation of ofatumumab is currently ongoing (ClinicalTrials.gov:NCT01457924). Importantly, this trial may also help to better define the dose range for ofatumumab as doses are much lower (3 mg, 30 mg, and 60 mg) than in the previous trial.

#### Targeting CD19 for B-cell depletion

In contrast to CD20, expression of CD19 is maintained on plasmablasts and subsets of PCs [54,132]. CD19, a member of the immunoglobulin domain-containing superfamily of transmembrane receptors, regulates the threshold for B-cell activation [133,134]. Relatively small changes in CD19 surface expression can thus lead to loss of tolerance and autoantibody production, which has been explored in transgenic (TG) mice that overexpress CD19. Furthermore, B-cell depletion in huCD19 TG mice with anti-CD19 reduced primary as well as secondary immune responses. Two weeks after treatment, IgM autoantibody levels significantly dropped below baseline values and the generation of IgG autoantibodies was almost completely inhibited [135]. These results suggest that targeting CD19 for B-cell depletion could indeed have a more pronounced effect on autoantibody production as compared with anti-CD20 mAbs.

MEDI-551 is an affinity-optimized and afucosylated mAb targeting CD19 (Table 1). MEDI-551 is specific for human CD19 and does not bind to mouse CD19 or CD19 from cynomologus monkeys. The lack of fucose results in approximately 10-fold increased affinity to FcγRIIIA and enhanced ADCC effector function [44]. In preclinical studies with huCD19 TG mice, MEDI-551 efficiently depleted blood and tissue B cells in a dose-dependent manner. In huCD19/huCD20 TG mice, MEDI-551 depleted B cells from blood and lymphoid organs at lower doses than rituximab, probably a result of the enhanced ADCC activity of the mAb [44]. MEDI-551 is currently undergoing evaluation in clinical trials (Table 1). A phase 1 safety and dose-finding study in patients with systemic sclerosis (ClinicalTrials.gov:NCT00946699) is still ongoing, and more recently a phase 1 trial in RRMS patients (ClinicalTrials.gov:NCT01585766) has been initiated. The hypothesis that targeting the CD19 antigen will have a greater impact on autoantibodies and that this could provide additional benefit to patients with autoimmune diseases will have to be tested in the current and future clinical trials.

#### Targeting the B-cell antigen CD22

CD22 belongs to the sialoadhesin (SIGLEC) subfamily of the immunoglobulin superfamily of cell surface receptors and specifically recognizes α-2,6-linked sialic acid on N-linked glycans. Some, but not all, functions of CD22 are controlled by ligand binding on the same cell surface (cis) or via cell-cell interactions (trans) [136,137]. CD22 is expressed on the majority of IgM^+^IgD^+ ^B cells, but expression is weak on germinal center B cells and absent on PCs [136,137]. While considered a B-cell antigen, CD22 has also been detected on human basophils as well as on conventional and plasmacytoid dendritic cells, although there appear to be clone-specific differences with regard to antibody reactivity [138-140]. CD22 plays an important regulatory role and is involved in the control of B-cell activation, peripheral B-cell homeostasis, survival, and cell cycle progression following activation [136].

Epratuzumab is the humanized IgG_1 _form of the murine CD22 mAb LL2, and exhibits modest ADCC but no CDC activity [141,142]. The relatively poor ADCC activity of epratuzumab is probably a result of the rapid internalization of CD22 following mAb binding [141,143]. While epratuzumab is able to trigger signaling events in B cells, as measured by tyrosine phosphorylation on the intracellular domain, the soluble form of the mAb has no profound impact on B-cell proliferation or apoptosis. When immobilized, however, epratuzumab does interfere with anti-IgM stimulated cell proliferation [142,143].

An early open-label phase 1/2 study tested the safety and potential for activity of epratuzumab in a small cohort of patients with primary Sjögren's syndrome [144]. In this trial, four infusions of 360 mg/m^2^, given every 2 weeks, appeared to be well tolerated and achieved clinical responses measured by a composite endpoint consisting of the Schirmer-I test, salivary flow, fatigue, erythrocyte sedimentation rate, and IgG. Of the treated patients, 53% achieved a 20% or greater improvement level at 6 weeks. Peripheral B-cell levels were reduced by 54% and 39% at 6 weeks and 18 weeks, respectively, while T cells and serum immunoglobulins did not change significantly [144]. An initial, open-label, phase 2 study in 14 patients with moderately active SLE (BILAG score 6 to 12) also produced promising results [145]. Patients again received four doses of 360 mg/m^2 ^epratuzumab every other week, together with acetaminophen and antihista mine as premedication, resulting in a significant improvement in the total BILAG score. However, while 77% of patients had a 50% or better improvement at week 6, this level of improvement was maintained in only 15% of patients by week 32. Similar to the primary Sjögren's syndrome study, peripheral blood B cells decreased by 35%, and this effect was maintained for the duration of the 6-month follow-up [145].

This initial study of epratuzumab in SLE was followed by two randomized controlled phase 3 trials - SL0003 (ClinicalTrials.gov:NCT00111306) and SL0004 (Clinical-Trials.gov:NCT00383214) - in moderate and severe SLE patients. Unfortunately both trials were interrupted due to limitations in drug supply. The available data from these trials were combined and presented at the EULAR in 2008. The combined results showed clinical meaningful efficacy, based on greater reduction in BILAG score and global disease assessment versus placebo. Also noted were improvements in health-related quality-of-life measures and clinically meaningful steroid sparing with both doses of epratuzumab tested [146-148].

Results from the EMBLEM phase 2b study (ClinicalTrials.gov:NCT00624351) were reported at the EULAR conference in 2010. Treatment with 600 mg epratuzumab weekly for 4 weeks resulted in greater BILAG improvements from BILAG A/B to BILAG D, compared with placebo. Overall, a cumulative epratuzumab dose of 2,400 mg resulted in clinically meaningful improvement with responder rates twice as high as in the placebo groups [149].

Two new phase 3 trials - EMBODY 1 (ClinicalTrials. gov:NCT01262365) and EMBODY 2 (ClinicalTrials.gov: NCT01261793) - were initiated in 2010 to confirm the activity of epratuzumab in patients with moderate to severe SLE (Table 1).

### Targeting B-cell survival factors and key cytokines

B-cell survival, differentiation, and functional properties are tightly regulated by a variety of cytokines and chemokines. Targeting survival and differentiation factors with specific mAbs or fusion proteins is an alternative approach to targeting B-cell surface antigens for active cell depletion.

In the mouse, B-cell activating factor (BAFF; also termed Blys), a TNF family member, has been shown to be an important survival signal for transitional B cells, but BAFF alone is not a survival signal for human transitional B cells in *in vitro *culture [150,151]. In clinical trials with Belimumab, however, *in vivo *BAFF blockade predominantly resulted in loss of transitional and naïve B cells, but not of memory B cells [152].

BAFF, as well as its close family member APRIL, have also been implicated in class switch recombination. Of note, APRIL appears to be a major driving source for IgA production and may play an important role in mucosal immunity [153]. Several other cytokines such as IL-10 and type I interferon have been reported to stimulate PC differentiation [154,155], but in humans IL-21 is the major cytokine responsible for maintaining the humoral immune response by induction of PC differentiation from both naive and memory B cells [156,157].

Splenic memory and germinal center B cells can be driven to differentiation by IL-10 and CD40L [154]. However, IL-21 is the most potent inducer of PC differentiation in the spleen [158-161]. BAFF also has the unique ability to substitute for CD40L in driving PC differentiation of human MZ B cells when co-stimulated with IL-21 [162]. BAFF has also been reported to co-stimulate PC differentiation of naive blood B cells in combination with IL-17, anti-CD40, and anti-IgM [163]. Although BAFF has been shown to be an important survival signal to PCs, it appears that April is one of the key survival factors for long-lived PCs in the bone marrow [164]. Of the many soluble factors involved in B-cell function and regulation, therapeutic approaches thus far have largely focused on the TNF family members BAFF and APRIL. More recently, IL-21 has gained significant attention owing its important role in the generation of PCs and antibody production.

#### Targeting B-cell activating factor (BlyS) with belimumab

The anti-BAFF mAb belimumab (HGS1006, LymphoStat-B) is the first biologic therapeutic to be approved for SLE, with approval in the United States, Canada, and Europe in 2011 [165]. Belimumab is a fully human IgG_1 _mAb that selectively inhibits BAFF, resulting in apoptosis of B cells [166]. The pivotal phase III trials with belimumab (BLISS-52 and BLISS-76) demonstrated a significant clinical response in patients with active, autoantibodypositive SLE who were receiving standard therapy [167-169]. In the BLISS-52 trial (ClinicalTrials.gov:NCT 00424476), a 52-week study, the rates of response (reduction ≥4 points on the SLE Response Index) at week 52 were 51% and 58% with belimumab 1 and 10 mg/kg/dose, respectively, versus 44% with placebo (*P *= 0.0129 and *P *= 0.006, respectively). In the BLISS-76 trial (Clinical- Trials.gov:NCT00410384), the rates of responses at week 52 were 40.6% and 43.2% with belimumab 1 and 10 mg/kg/dose versus 33.5% with placebo (*P *= NS and *P *= 0.02); at 76 weeks, however, response rates were 39.1% and 38.5% with belimumab 1 and 10 mg/kg/dose versus 32.4% with placebo, thus not reaching statistical significance (*P *= 0.11 and *P *= 0.13). The tolerability data from these studies did not suggest any overall differences between belimumab and placebo [170].

In a long-term phase II study, circulating total B cells were significantly decreased at 52 weeks of therapy. Newly activated B cells were reduced at 12 to 24 weeks, and plasmablasts and pre-switched memory cells at 76 weeks. Post-switched memory cells were not significantly decreased [170]. Thus, belimumab appears to affect newly activated B lymphocytes more than memory B lymphocytes or PCs. Belimumab use was also associated with decreases in total IgM concentrations (*P *<0.05) but was not associated with significant changes in total IgG, anti-DNA IgM, anti-DNA IgG, or T lymphocytes.

Additional large phase III trials to evaluate long-term safety are currently active, and a phase III trial has been initiated to further investigate and validate the subcutaneous route of administration. A phase III multicenter, open-label, parallel study (ClinicalTrials.gov: NCT00712933) to assess the safety and efficacy (SLICC/ ACR damage index) is expected to be completed by March 2015. Another phase III trial (ClinicalTrials.gov: NCT01345253) is in progress to evaluate the efficacy and safety of belimumab in a Northeast Asian population. Additional trials will evaluate vaccine responses post belimumab (ClinicalTrials.gov:NCT01597492) and assess activity specifically in black race patients (EMBRACE, ClinicalTrials.gov:NCT01632241).

Additional indications are under investigation for belimumab either through investigator-sponsored or company-sponsored trials (Table 1). These indications include symptomatic Waldenstrom's macroglobulinemia (ClinicalTrials.gov:NCT01142011), idiopathic membranous glomerulonephropathy (ClinicalTrials.gov:NCT01610492), Sjögren's syndrome (ClinicalTrials.gov:NCT01160666, ClinicalTrials.gov:NCT01008982), prevention of kidney transplant rejection (ClinicalTrials.gov:NCT01536379), chronic immune thrombocytopenia (ClinicalTrials.gov: NCT01440361), and myasthenia gravis (ClinicalTrials. gov:NCT01480596). These phase II trials are all expected to report data in the next 1 to 2 years.

Prior to approval in SLE, the inventor company also conducted a phase II trial in RA patients. In this trial the ACR20 response was significantly higher with 1 mg/kg versus placebo (35% vs. 16%), but ACR20 responses achieved with the 4 mg/kg and 10 mg/kg doses (25% and 28%) did not reach statistical significance and the extension trial was subsequently terminated.

#### Tabalumab anti-B-cell activating factor

Another mAb targeting BAFF is being pursued for the treatment of SLE and RA. Tabalumab (LY-2127399) is a humanized IgG_4 _antibody designed to neutralize soluble and membrane-bound BAFF. Given the similar mechanism of action and the proven clinical and regulatory success of belimumab, it was decided to skip phase II trials and move directly towards phase III trials in SLE (ClinicalTrials.gov:NCT01196091, ClinicalTrials.gov:NCT01205438) [171]. Tabalumab is also being developed for RA, with multiple phase 3 trials ongoing (FLEX-M, FLEX-O, and FLEX-V), as well as an open-label phase 3b trial for evaluation of long-term safety. The proof-of-concept phase 2 trial (ClinicalTrials.gov:NCT01576549) is also still ongoing, although the primary endpoint at 24 weeks provided sufficient evidence to move into the phase 3 trials mentioned above. Additionally, a phase 2 trial in RRMS was conducted recently (ClinicalTrials. gov:NCT00882999), although it does not appear that this indication is currently being pursued.

#### Blockade of B-cell activating factor and April with atacicept

An alternative approach for targeting BAFF has been pursued. Atacicept (TACI-Ig) is a fusion protein comprised of the extracellular domain of transmembrane activator and calcium-modulating ligand interactor fused to the Fc of human IgG_1_, and binds and blocks both BAFF and APRIL to inhibit B-cell maturation [172]. Atacicept has been studied in phase Ib trials in SLE patients, using both the intravenous and subcutaneous route of administration [173-175]. Dose-dependent reductions in B lymphocytes and immunoglobulin levels were observed. More recently, atacicept is being evaluated in a double-blind, placebo-controlled, multicenter phase 2/3 trial to determine the most effective dose of atacicept for reducing flares (ClinicalTrials.gov:NCT00624338), although it appears that the higher dose of 150 mg subcutaneously was deemed to have an unfavorable risk/benefit profile and is no longer enrolling patients (Table 1).

Previously, trials were conducted in lupus nephritis patients; however, these trials were terminated due to safety concerns. The use of atacicept in RA patients has also been investigated in several phase 2 trials (ClinicalTrials.gov:NCT00595413, ClinicalTrials.gov: NCT-00430495, ClinicalTrials.gov:NCD00664521). Published results indicate that atacicept was no better than placebo in the treatment of RA, although a dose-dependent reduction in serum rheumatoid factor levels as well as reductions in circulating B cells and PCs were noted [176,177]. Moreover, clinical trials of atacicept in MS patients (phase 2, ClinicalTrials.gov:NCT00642902) and optic neuritis patients (phase 2, ClinicalTrials.gov: NCT00624468) were terminated because atacicept worsened disease activity for MS patients. The results in the MS trial and the disconnect between the biological and clinical response in RA clearly came as a surprise, given that B-cell depletion with CD20 mAbs is effective in both of these indications. One has to keep in mind, however, that atacicept and rituximab target different B-cell populations, with the latter having a broad depletion pattern. One possible explanation could be that atacicept has a significant impact on Bregs without sufficiently depleting pathogenic subsets. Bregs have been implicated in modulating the response to B-cell depletion in experimental autoimmune encephalomyelitis and other models, and are discussed in detail in the accompanying article by Tedder and colleagues (this issue).

Interestingly, Fernandez and colleagues recently found that overexpression of APRIL in a mouse model of collagen-induced arthritis ameliorates rather than promotes disease [178]. This effect was correlated with decreases in collagen-specific autoantibody titers and immune complex deposition, suggesting that ectopic April expression negatively regulates antibody responses. Further, April TG mice had increased frequencies of IL-10-producing B cells along with decreased numbers of memory B cells in the spleen and PCs in the bone marrow. One should note that transmembrane activator and calcium-modulating ligand interactor (TACI) is also expressed on monocytes and dendritic cells, and BAFF receptor is expressed on activated T cells and regulatory T cells. T-cell responses and polarization towards Th1, Th2, or Th17 cells, however, were not noticeably affected in APRIL TG mice [178]. Clearly, the requirement of APRIL for the generation and/or maintenance of IL-10- producing B cells as well as the effect of BAFF/APRIL neutralization on non-B cells require further investigation.

#### Targeting IL-21 with mAb NNC114-0005

As outlined above, IL-21, a member of the gamma chain receptor cytokine family, plays a central role in the humoral immune response and in the production of autoantibodies. In addition, IL-21 is involved in the regulation of other immune functions, including CD4 T-cell differentiation and expansion, CD8 T-cell and natural killer cell expansion and cytolytic activity, as well as others [179]. IL-21 has now been linked to several human autoimmune pathologies including SLE, psoriasis, Sjögren's syndrome, MS and RA, making this cytokine an attractive target for therapeutic intervention [160,180-186].

A panel of high-affinity (sub pM K_D_) fully human anti-IL21 mAbs was recently described in the literature [187]. The therapeutic candidate mAb NNC114-0005 (NN8828), derived from this panel, is currently undergoing early clinical testing. A first phase 1 study testing safety, tolerability, pharmacokinetics, and pharmacodynamics of various doses after intravenous or subcutaneous administration to healthy volunteers and RA patients has been completed (ClinicalTrials.gov:NCT01208506). A second phase 1 RA trial testing repeat administration of the mAb via subcutaneous injection is ongoing (Table 1).

### Blockade of co-stimulatory pathways

Adaptive immune responses are shaped and controlled by co-stimulatory receptor-ligand pairs, which are classified into several families. Co-stimulatory molecules of the immunoglobulin superfamily include the CD28/ CD80/CD86 (B7.1/B7.2), CTLA4/CD80/CD86 (B7.1/B7.2), PD-1/PDL, and ICOS/B7RP-1 (ICOS-L) pathways. The co-stimulatory CD40/CD40L (CD154), OX40/OX40 ligand, and GITR/GITR ligand receptor/ligand pairs belong to the TNF/TNF receptor superfamily [188]. Costimulatory signals triggered by interaction with antigenpresenting cells are critical for an effective T-cell response. B cells can also present antigen to T cells and receive costimulatory signals in return. These signals are important for humoral immune responses by promoting germinal center reaction and PC differentiation. Therapeutic approaches aimed at interfering with B-cell responses by blocking co-stimulatory molecules have largely focused on two pathways, ICOS/B7RP-1 and CD40/CD40L.

ICOS-deficient patients are characterized by a severe reduction in the number of B lymphocytes and absence of memory switched B cells and PCs [189]. Defects in germinal center formation and class switch recombination have also been reported in ICOS and ICOS ligand (B7RP-1) deficient mice, underscoring the importance of this signaling axis [190].

CD40 (TNFRSF5) is a cell surface receptor on B cells, monocytes, macrophages, dendritic cells, eosinophils, and activated CD8 T cells [191]. The ligand for CD40, CD40L (CD154, TNFSF5), is transiently expressed on activated CD4, CD8, and γδ T cells, as well as on activated B cells, activated platelets, dendritic cells, smooth muscle cells, vascular endothelial cells, and epithelial cells. The broad expression of both receptor and ligand emphasize the important but also complex role this pathway plays in the regulation of immune responses. Mutations in the CD40L gene are the cause of the X-linked hyper-IgM syndrome, a disease characterized by an overabundance of IgM in the serum and a lack of IgG, IgE, and IgA [192]. In addition, mutations in CD40L have been identified in a subset of patients with common variable immunodeficiency, which is characterized by a defect in B-cell differentiation and hypogammaglobulinemia [193]. These observations as well as an abundance of data from mouse models clearly establish CD40/ CD40L as central pathway required for humoral immune responses. Further, serum levels of soluble CD40L have been found to be elevated in a variety of autoimmune conditions, including SLE, RA, Sjögren's syndrome, and inflammatory bowel disease, and were correlated to autoantibody titers and disease activity [191,194].

#### Blocking B7RP-1 (ICOSL) with AMG-557

AMG-557 is a fully human IgG_2 _mAb directed against the ICOS ligand B7RP-1 [195]. Preclinical studies demonstrated that treatment with blocking anti-B7RP-1 mAbs has activity in several mouse models of inflammatory and autoimmune disease. For example, in the NZB/NZW F1 mouse lupus model, blockade of B7RP-1 reduced serum titers of anti-dsDNA autoantibodies, the incidence of proteinuria, and significantly increased survival [196]. Similarly, treatment with anti-B7RP-1 mAb reduced disease activity in the collagen-induced arthritis model. Blockade of the ICOS/B7RP-1 pathway with anti-B7RP-1 also inhibited T-cell activation and reduced the numbers of T-follicular helper cells as well as germinal center B cells [196]. The anti-human-B7RP-1 mAb AMG-557 is in early stage clinical testing. The first study initiated was a dose escalation trial in SLE patients, positive for antinuclear antigen autoantibodies (ClinicalTrials.gov:NCT00774943). In addition, phase 1 trials in patients with subacute cutaneous lupus erythematosus and psoriasis are ongoing. Results from these clinical studies are not yet available (Table 1).

#### Targeting ICOS-expressing T cells with MEDI-570

MEDI-570 is a human IgG_1 _mAb directed against ICOS and was generated from the human anti-human-ICOS IgG_2κ _mAb JTA-009 [197]. Similar to MEDI-551, MEDI-570 has enhanced ADCC effector function, due to the lack of fucose from the Fc carbohydrate. In cynomologus monkeys, MEDI-570 resulted in a dose-dependent and reversible depletion of ICOS^+ ^T cells in blood and secondary lymphoid organs. Consistent with the depletion of T-follicular helper, splenic germinal centers were reduced in size and frequency, which was accompanied by a significant reduction of germinal center B cells. This effect was reversible, with new germinal centers forming 30 days after MEDI-570 administration, indicative of follicular reconstitution [198]. MEDI-570 is being evaluated as a treatment for SLE, based on depletion of ICOS^+ ^T cells, with loss of the effector cytokines and loss of support for germinal center formation and reduction in autoantibody production. A phase 1 single ascending dose trial in patients with moderate to severe SLE is ongoing (ClinicalTrials.gov:NCT01127321) (Table 1).

#### Blockade of CD40 ligand with CDP7657

Several blocking mAbs targeting CD40L were developed, and two of these - ruplizumab (BG9588, hu5c8) and toralizumab (IDEC-131, hu24-31) - were evaluated in phase 1 and phase 2 clinical trials. Some of these trials, such as the phase 2 trial of ruplizumab in lupus nephritis and a phase 1 trial of toralizumab in ITP, showed some early but promising signs of clinical activity [191]. Further clinical development of these mAbs, however, was suspended due to thromboembolic events observed in some of the treated patients. Similarly, development of the anti-CD40L mAb ABI793 was stopped after thromboembolisms were observed in preclinical studies in nonhuman primates. A probable explanation for this complication, which appears to be a class effect, is the formation of platelet aggregates by binding of the IgG_1 _mAbs to CD40L on the platelet surface and simultaneous engagement of the FcγR CD32A, which is also expressed on platelets, by the mAb Fc. This hypothesis is supported by recent findings with anti-CD40L mAbs in a human CD32A TG mouse model [199]. New approaches are being developed to target and neutralize CD40L, while avoiding platelet-dependent adverse effects. One such approach has already entered the clinic.

CDP7657 is an anti-CD40L Fab fragment under development for the treatment of SLE [200]. The monovalent nature of the molecule and lack of the mAb Fc are designed to minimize or eliminate the risk for platelet aggregation and thromboembolisms observed with the anti-CD40L mAbs in IgG_1 _format. CDP7657 is pegylated to increase the halflife of the low molecular weight molecule. In a preclinical humanized SCID mouse model, CDP7657 demonstrated dose-dependent inhibition of antibody responses to tetanus toxoid. CDP7657 also inhibited primary and secondary immune responses to tetanus toxoid in a nonhuman primate mode [200]. CDP7657 is currently being evaluated in a single-dose phase 1 study conducted in healthy volunteers and SLE patients (ClinicalTrials.gov:NCT01093911) (Table 1).

### Effects beyond B cells

The success of rituximab has resulted in significant interest in targeting CD20, leading to this variety of anti-CD20 mAbs with different functional properties. However, based on CD20 expression along the B-cell lineage, all of these mAbs are expected to deplete B-cell subsets in blood and tissues similar to rituximab. Importantly, antibody-secreting PCs do not express CD20 and are therefore not directly targeted for depletion by rituximab and other CD20 mAbs [54,132]. The lack of a direct impact on PCs may explain the inconsistent effects of rituximab on various autoantibodies [54,201]. While rheumatoid factor levels in RA and anti-dsDNA autoantibodies can drop significantly after B-cell depletion therapy, other autoantibodies are only modestly affected or not at all. One possible explanation for this is that some autoantibodies are generated by short-lived PCs, which have a survival span of only a few weeks, and will be eliminated over time in the absence of a replenishing B-cell pool. However, another suggestion is that short-lived PCs still express CD20 levels that are sufficient for depletion by rituximab, based on results from a study conducted in a human CD20 TG mouse model [202]. Whether this latter mechanism is indeed effective in human patients remains unclear. In either case, autoantibody-secreting PCs will still persist in survival niches in the bone marrow or inflamed tissues, contributing to disease flares or symptoms [203].

The relevance of PCs in disease is further emphasized by the finding that elevated PC numbers at baseline predict poor or nonresponse to rituximab and ocrelizumab in RA [62,204]. The concept of targeting PCs in autoimmune diseases is beginning to be explored in the clinic. The proteasome inhibitor bortezomib, which eliminates PCs and is approved for the treatment of multiple myeloma, has proven efficacious in an experimental model of SLE clinically and by significantly decreasing autoantibody titers in preventive and therapeutic approaches [205]. At the 2012 EULAR conference, Hiepe and colleagues reported on a small study with bortezomib in treatment refractory SLE patients. Bortezomib significantly decreased disease activity scores and serum anti-dsDNA autoantibody titers. Patients with lupus nephritis also experienced a decline in proteinuria [206]. These results further support the rationale for direct or indirect targeting of PCs, which provide additional benefit to patients, provided that any such approach is not associated with an unacceptable safety profile.

Autoimmune diseases are complex in their origin and pathobiology, however, and several other cell types - such as CD4 and CD8 T cells, macrophages, dendritic cells as well as others - have been implicated. The relative contribution of B cells and PCs versus other cell types, and the relevance of regulatory cells such as regulatory T cells and Bregs, may also vary between different indications. In addition, as outlined above, B cells may influence other inflammatory cells through the production of cytokines, antigen presentation and other mechanisms. Interestingly, a variety of diseases that have traditionally been considered to be T-cell-mediated, such as RRMS, do respond well to B-cell depletion therapy. Several clinical studies have investigated the impact of B-cell depletion on some of these key cell populations. For example, B-cell depletion with rituximab resulted not only in near-complete depletion of B cells from the cerebrospinal fluid of MS patients, but also in an approximately 50% reduction of T cells [207,208]. Further, in RA patients, rituximab reduced Th17 cells from synovial tissue, which was correlated with better clinical outcome [209]. This study, however, did not find significant effects of rituximab on regulatory T cells, Th1 cells, or TNFα responses. The effect of B-cell depletion on T cells was also investigated in SLE patients treated with rituximab. Here, rituximab resulted in depletion of naïve and memory B cells from blood, along with substantial reductions in CD40L^+ ^and ICOS^+ ^CD4 T cells [58]. One should note, however, that other B-cell targeted therapies have more restricted (or selective) effects on B cells, and may therefore differ significantly with regard to their effects on other inflammatory cells - but this has not yet been studied in detail in patients. How varying effects on non-B-cell populations will influence efficacy of different B-cell targeted therapeutics in various indications will have to be determined in ongoing and future clinical trials.

The various strategies pursued to target B cells are clearly not interchangeable and we have to learn how to best tailor the therapy to the condition. Results from the ongoing clinical trials with B-cell targeted biologics spanning a wide array of mechanisms will greatly increase our understanding of autoimmune diseases and will provide the basis for optimally matching the therapeutic approach to a given patient population to improve clinical efficacy while minimizing potential side effects.

## Abbreviations

ACR: American College of Rheumatology; ADCC: antibody-dependent cellular cytotoxicity; AIHA: autoimmune hemolytic anemia; APRIL: a proliferation-inducing ligand; BAFF: B-cell activating factor; BCR: B-cell receptor; BILAG: British Isles Lupus Assessment Group; Breg: regulatory B cell; CDC: complement-dependent cytotoxicity; CD40L: CD40 ligand; dsDNA: double-stranded DNA; Dsg: desmoglein; EULAR: annual European Congress of Rheumatology; EXPLORER: Exploratory Phase II/III SLE Evaluation of Rituximab; FcγR: Fcγ receptor; IFN: interferon; IL: interleukin; ITP: immune thrombocytopenia; LUNAR: Lupus Nephritis Assessment with Rituximab; mAb: monoclonal antibody; MHC: major histocompatibility complex; MS: multiple sclerosis; NF: nuclear factor; PC: plasma cell; PML: progressive multifocal leukoencephalopathy; RA: rheumatoid arthritis; RRMS: relapsing remitting multiple sclerosis; SLE: systemic lupus erythematosus; TEARS: Tolerance and Efficacy of Rituximab in Sjogren's Disease; TG: transgenic; Th: T-helper; TNF: tumor necrosis factor.

## Competing interests

KM, RE and RH are full-time employees of MedImmune, LLC. The remaining authors declare that they have no competing interests.

## References

[B1] Slifka MK Antia R Whitmire JK Ahmed R Humoral immunity due to long-lived plasma cells Immunity 1998 8 363 372 9529153 10.1016/s1074-7613(00)80541-5

[B2] Obukhanych TV Nussenzweig MC T-independent type II immune responses generate memory B cells J Exp Med 2006 203 305 310 16476769 10.1084/jem.20052036 PMC2118207

[B3] Pillai S Cariappa A Moran ST Positive selection and lineage commitment during peripheral B-lymphocyte development Immunol Rev 2004 197 206 218 14962197 10.1111/j.0105-2896.2003.097.x

[B4] Anolik JH Looney RJ Lund FE Randall TD Sanz I Insights into the heterogeneity of human B cells: diverse functions, roles in autoimmunity, and use as therapeutic targets Immunol Res 2009 45 144 158 19350211 10.1007/s12026-009-8096-7 PMC2891332

[B5] Berland R Wortis HH Origins and functions of B-1 cells with notes on the role of CD5 Annu Rev Immunol 2002 20 253 300 11861604 10.1146/annurev.immunol.20.100301.064833

[B6] Hayakawa K Hardy RR Honda M Herzenberg LA Steinberg AD Herzenberg LA Ly-1 B cells: functionally distinct lymphocytes that secrete IgM autoantibodies Proc Natl Acad Sci USA 1984 81 2494 2498 6609363 10.1073/pnas.81.8.2494 PMC345088

[B7] Hayakawa K Hardy RR Normal, autoimmune, and malignant CD5^+ ^B cells: the Ly-1 B lineage? Annu Rev Immunol 1988 6 197 218 3289567 10.1146/annurev.iy.06.040188.001213

[B8] Hardy RR Hayakawa K Shimizu M Yamasaki K Kishimoto T Rheumatoid factor secretion from human Leu-1^+ ^B cells Science 1987 236 81 83 3105057 10.1126/science.3105057

[B9] Casali P Burastero SE Nakamura M Inghirami G Notkins AL Human lymphocytes making rheumatoid factor and antibody to ssDNA belong to Leu-1^+ ^B-cell subset Science 1987 236 77 81 3105056 10.1126/science.3105056

[B10] Baumgarth N The double life of a B-1 cell: self-reactivity selects for protective effector functions Nat Rev Immunol 2011 11 34 46 21151033 10.1038/nri2901

[B11] Lanzavecchia A Antigen-specific interaction between T and B cells Nature 1985 314 537 539 3157869 10.1038/314537a0

[B12] Rodriguez-Pinto D B cells as antigen presenting cells Cell Immunol 2005 238 67 75 16574086 10.1016/j.cellimm.2006.02.005

[B13] Chen X Jensen PE The role of B lymphocytes as antigen-presenting cells Arch Immunol Ther Exp (Warsz) 2008 56 77 83 18373241 10.1007/s00005-008-0014-5

[B14] Fearon DT Carter RH The CD19/CR2/TAPA-1 complex of B lymphocytes: linking natural to acquired immunity Annu Rev Immunol 1995 13 127 149 7542009 10.1146/annurev.iy.13.040195.001015

[B15] Dal Porto JM Gauld SB Merrell KT Mills D Pugh-Bernard AE Cambier J B cell antigen receptor signaling 101 Mol Immunol 2004 41 599 613 15219998 10.1016/j.molimm.2004.04.008

[B16] Zhong G Reis e Sousa C Germain RN Antigen-unspecific B cells and lymphoid dendritic cells both show extensive surface expression of processed antigen-major histocompatibility complex class II complexes after soluble protein exposure in vivo or in vitro J Exp Med 1997 186 673 682 9271583 10.1084/jem.186.5.673 PMC2199022

[B17] Cassell DJ Schwartz RH A quantitative analysis of antigen-presenting cell function: activated B cells stimulate naive CD4 T cells but are inferior to dendritic cells in providing costimulation J Exp Med 1994 180 1829 1840 7525839 10.1084/jem.180.5.1829 PMC2191739

[B18] Yan J Wolff MJ Unternaehrer J Mellman I Mamula MJ Targeting antigen to CD19 on B cells efficiently activates T cells Int Immunol 2005 17 869 877 15967786 10.1093/intimm/dxh266

[B19] Morris SC Lees A Finkelman FD In vivo activation of naive T cells by antigen-presenting B cells J Immunol 1994 152 3777 3785 8144947

[B20] Bouaziz JD Yanaba K Venturi GM Wang Y Tisch RM Poe JC Tedder TF Therapeutic B cell depletion impairs adaptive and autoreactive CD4^+ ^T cell activation in mice Proc Natl Acad Sci USA 2007 104 20878 20883 18093919 10.1073/pnas.0709205105 PMC2409235

[B21] O'Neill SK Shlomchik MJ Glant TT Cao Y Doodes PD Finnegan A Antigenspecific B cells are required as APCs and autoantibody-producing cells for induction of severe autoimmune arthritis J Immunol 2005 174 3781 3788 15749919 10.4049/jimmunol.174.6.3781

[B22] Takemura S Klimiuk PA Braun A Goronzy JJ Weyand CM T cell activation in rheumatoid synovium is B cell dependent J Immunol 2001 167 4710 4718 11591802 10.4049/jimmunol.167.8.4710

[B23] Lund FE Cytokine-producing B lymphocytes-key regulators of immunity Curr Opin Immunol 2008 20 332 338 18417336 10.1016/j.coi.2008.03.003 PMC2474694

[B24] Pistoia V Production of cytokines by human B cells in health and disease Immunol Today 1997 18 343 350 9238838 10.1016/s0167-5699(97)01080-3

[B25] Wagner M Poeck H Jahrsdoerfer B Rothenfusser S Prell D Bohle B Tuma E Giese T Ellwart JW Endres S Hartmann G IL-12p70-dependent Th1 induction by human B cells requires combined activation with CD40 ligand and CpG DNA J Immunol 2004 172 954 963 14707068 10.4049/jimmunol.172.2.954

[B26] Tumanov AV Kuprash DV Mach JA Nedospasov SA Chervonsky AV Lymphotoxin and TNF produced by B cells are dispensable for maintenance of the follicle-associated epithelium but are required for development of lymphoid follicles in the Peyer's patches J Immunol 2004 173 86 91 15210762 10.4049/jimmunol.173.1.86

[B27] Barr TA Shen P Brown S Lampropoulou V Roch T Lawrie S Fan B O'Connor RA Anderton SM Bar-Or A Fillatreau S Gray D B cell depletion therapy ameliorates autoimmune disease through ablation of IL-6-producing B cells J Exp Med 2012 209 1001 1010 22547654 10.1084/jem.20111675 PMC3348102

[B28] Onal M Xiong J Chen X Thostenson JD Almeida M Manolagas SC O'Brien CA RANKL expression by B lymphocytes contributes to ovariectomyinduced bone loss J Biol Chem 2012 in press 10.1074/jbc.M112.377945 PMC3436192 22782898

[B29] Heider U Zavrski I Jakob C Bangeroth K Fleissner C Langelotz C Possinger K Hofbauer LC Viereck V Sezer O Expression of receptor activator of NF-kappaB ligand (RANKL) mRNA in human multiple myeloma cells J Cancer Res Clin Oncol 2004 130 469 474 15205949 10.1007/s00432-004-0578-3 PMC12161873

[B30] Yeo L Toellner KM Salmon M Filer A Buckley CD Raza K Scheel-Toellner D Cytokine mRNA profiling identifies B cells as a major source of RANKL in rheumatoid arthritis Ann Rheum Dis 2011 70 2022 2028 21742639 10.1136/ard.2011.153312 PMC3184241

[B31] Jimenez-Boj E Redlich K Turk B Hanslik-Schnabel B Wanivenhaus A Chott A Smolen JS Schett G Interaction between synovial inflammatory tissue and bone marrow in rheumatoid arthritis J Immunol 2005 175 2579 2588 16081832 10.4049/jimmunol.175.4.2579

[B32] Fillatreau S Sweenie CH McGeachy MJ Gray D Anderton SM B cells regulate autoimmunity by provision of IL-10 Nat Immunol 2002 3 944 950 12244307 10.1038/ni833

[B33] Mizoguchi A Mizoguchi E Smith RN Preffer FI Bhan AK Suppressive role of B cells in chronic colitis of T cell receptor alpha mutant mice J Exp Med 1997 186 1749 1756 9362534 10.1084/jem.186.10.1749 PMC2199135

[B34] Mauri C Gray D Mushtaq N Londei M Prevention of arthritis by interleukin 10-producing B cells J Exp Med 2003 197 489 501 12591906 10.1084/jem.20021293 PMC2193864

[B35] Tian J Zekzer D Hanssen L Lu Y Olcott A Kaufman DL Lipopolysaccharideactivated B cells down-regulate Th1 immunity and prevent autoimmune diabetes in nonobese diabetic mice J Immunol 2001 167 1081 1089 11441119 10.4049/jimmunol.167.2.1081

[B36] Duddy M Niino M Adatia F Hebert S Freedman M Atkins H Kim HJ Bar-Or A Distinct effector cytokine profiles of memory and naive human B cell subsets and implication in multiple sclerosis J Immunol 2007 178 6092 6099 17475834 10.4049/jimmunol.178.10.6092

[B37] Nimmerjahn F Ravetch JV Fcγ receptors as regulators of immune responses Nat Rev Immunol 2008 8 34 47 18064051 10.1038/nri2206

[B38] Glennie MJ French RR Cragg MS Taylor RP Mechanisms of killing by anti- CD20 monoclonal antibodies Mol Immunol 2007 44 3823 3837 17768100 10.1016/j.molimm.2007.06.151

[B39] Cartron G Dacheux L Salles G Solal-Celigny P Bardos P Colombat P Watier H Therapeutic activity of humanized anti-CD20 monoclonal antibody and polymorphism in IgG Fc receptor FcγRIIIa gene Blood 2002 99 754 758 11806974 10.1182/blood.v99.3.754

[B40] Weng WK Levy R Two immunoglobulin G fragment C receptor polymorphisms independently predict response to rituximab in patients with follicular lymphoma J Clin Oncol 2003 21 3940 3947 12975461 10.1200/JCO.2003.05.013

[B41] Anolik JH Campbell D Felgar RE Young F Sanz I Rosenblatt J Looney RJ The relationship of FcγRIIIa genotype to degree of B cell depletion by rituximab in the treatment of systemic lupus erythematosus Arthritis Rheum 2003 48 455 459 12571855 10.1002/art.10764

[B42] Desjarlais JR Lazar GA Zhukovsky EA Chu SY Optimizing engagement of the immune system by anti-tumor antibodies: an engineer's perspective Drug Discov Today 2007 12 898 910 17993407 10.1016/j.drudis.2007.08.009

[B43] Shields RL Lai J Keck R O'Connell LY Hong K Meng YG Weikert SH Presta LG Lack of fucose on human IgG_1 _N-linked oligosaccharide improves binding to human FcγRIII and antibody-dependent cellular toxicity J Biol Chem 2002 277 26733 26740 11986321 10.1074/jbc.M202069200

[B44] Herbst R Wang Y Gallagher S Mittereder N Kuta E Damschroder M Woods R Rowe DC Cheng L Cook K Evans K Sims GP Pfarr DS Bowen MA Dall'Acqua W Shlomchik M Tedder TF Kiener P Jallal B Wu H Coyle AJ B-cell depletion in vitro and in vivo with an afucosylated anti-CD19 antibody J Pharmacol Exp Ther 2010 335 213 222 20605905 10.1124/jpet.110.168062

[B45] Stavenhagen JB Gorlatov S Tuaillon N Rankin CT Li H Burke S Huang L Vijh S Johnson S Bonvini E Koenig S Fc optimization of therapeutic antibodies enhances their ability to kill tumor cells in vitro and controls tumor expansion in vivo via low-affinity activating Fcgamma receptors Cancer Res 2007 67 8882 8890 17875730 10.1158/0008-5472.CAN-07-0696

[B46] Lazar GA Dang W Karki S Vafa O Peng JS Hyun L Chan C Chung HS Eivazi A Yoder SC Vielmetter J Carmichael DF Hayes RJ Dahiyat BI Engineered antibody Fc variants with enhanced effector function Proc Natl Acad Sci USA 2006 103 4005 4010 16537476 10.1073/pnas.0508123103 PMC1389705

[B47] Masuda K Kubota T Kaneko E Iida S Wakitani M Kobayashi-Natsume Y Kubota A Shitara K Nakamura K Enhanced binding affinity for FcγRIIIa of fucose-negative antibody is sufficient to induce maximal antibody-dependent cellular cytotoxicity Mol Immunol 2007 44 3122 3131 17379311 10.1016/j.molimm.2007.02.005

[B48] Zhou X Hu W Qin X The role of complement in the mechanism of action of rituximab for B-cell lymphoma: implications for therapy Oncologist 2008 13 954 966 18779537 10.1634/theoncologist.2008-0089

[B49] van Oers MH Van Glabbeke M Giurgea L Klasa R Marcus RE Wolf M Kimby E van t Veer M Vranovsky A Holte H Hagenbeek A Rituximab maintenance treatment of relapsed/resistant follicular non-Hodgkin's lymphoma: longterm outcome of the EORTC 20981 phase III randomized intergroup study J Clin Oncol 2010 28 2853 2858 20439641 10.1200/JCO.2009.26.5827 PMC2903319

[B50] Molina A A decade of rituximab: improving survival outcomes in non-Hodgkin's lymphoma Annu Rev Med 2008 59 237 250 18186705 10.1146/annurev.med.59.060906.220345

[B51] Stone JH Merkel PA Spiera R Seo P Langford CA Hoffman GS Kallenberg CG St Clair EW Turkiewicz A Tchao NK Webber L Ding L Sejismundo LP Mieras K Weitzenkamp D Ikle D Seyfert-Margolis V Mueller M Brunetta P Allen NB Fervenza FC Geetha D Keogh KA Kissin EY Monach PA Peikert T Stegeman C Ytterberg SR Specks U RAVE-ITN Research Group Rituximab versus cyclophosphamide for ANCA-associated vasculitis N Engl J Med 2010 363 221 232 20647199 10.1056/NEJMoa0909905 PMC3137658

[B52] Jones RB Tervaert JW Hauser T Luqmani R Morgan MD Peh CA Savage CO Segelmark M Tesar V van Paassen P Walsh D Walsh M Westman K Jayne DREuropean Vasculitis Study Group Rituximab versus cyclophosphamide in ANCA-associated renal vasculitis N Engl J Med 2010 363 211 220 20647198 10.1056/NEJMoa0909169

[B53] Pitashny M Shoenfeld Y B cell depletion in autoimmune rheumatic diseases Autoimmun Rev 2005 4 436 441 16137609 10.1016/j.autrev.2005.03.002

[B54] Levesque MC St Clair EW B cell-directed therapies for autoimmune disease and correlates of disease response and relapse J Allergy Clin Immunol 2008 121 13 21 quiz 22-23 18206502 10.1016/j.jaci.2007.11.030

[B55] Leandro MJ Cambridge G Ehrenstein MR Edwards JC Reconstitution of peripheral blood B cells after depletion with rituximab in patients with rheumatoid arthritis Arthritis Rheum 2006 54 613 620 16447239 10.1002/art.21617

[B56] Anolik JH Friedberg JW Zheng B Barnard J Owen T Cushing E Kelly J Milner EC Fisher RI Sanz I B cell reconstitution after rituximab treatment of lymphoma recapitulates B cell ontogeny Clin Immunol 2007 122 139 145 17008130 10.1016/j.clim.2006.08.009

[B57] Abdulahad WH Meijer JM Kroese FG Meiners PM Vissink A Spijkervet FK Kallenberg CG Bootsma H B cell reconstitution and T helper cell balance after rituximab treatment of active primary Sjogren's syndrome: a double-blind, placebo-controlled study Arthritis Rheum 2011 63 1116 1123 21225693 10.1002/art.30236

[B58] Iwata S Saito K Tokunaga M Yamaoka K Nawata M Yukawa S Hanami K Fukuyo S Miyagawa I Kubo S Tanaka Y Phenotypic changes of lymphocytes in patients with systemic lupus erythematosus who are in longterm remission after B cell depletion therapy with rituximab J Rheumatol 2011 38 633 641 21159836 10.3899/jrheum.100729

[B59] Roll P Palanichamy A Kneitz C Dorner T Tony HP Regeneration of B cell subsets after transient B cell depletion using anti-CD20 antibodies in rheumatoid arthritis Arthritis Rheum 2006 54 2377 2386 16869000 10.1002/art.22019

[B60] Palanichamy A Barnard J Zheng B Owen T Quach T Wei C Looney RJ Sanz I Anolik JH Novel human transitional B cell populations revealed by B cell depletion therapy J Immunol 2009 182 5982 5993 19414749 10.4049/jimmunol.0801859 PMC2746373

[B61] Sellam J Rouanet S Hendel-Chavez H Abbed K Sibilia J Tebib J Le Loet X Combe B Dougados M Mariette X Taoufik Y Blood memory B cells are disturbed and predict the response to rituximab in patients with rheumatoid arthritis Arthritis Rheum 2011 63 3692 3701 22127692 10.1002/art.30599

[B62] Vital EM Dass S Rawstron AC Buch MH Goeb V Henshaw K Ponchel F Emery P Management of nonresponse to rituximab in rheumatoid arthritis: predictors and outcome of re-treatment Arthritis Rheum 2010 62 1273 1279 20131284 10.1002/art.27359

[B63] Leandro MJ Becerra-Fernandez E B-cell therapies in established rheumatoid arthritis Best Pract Res Clin Rheumatol 2011 25 535 548 22137923 10.1016/j.berh.2011.10.005

[B64] Buch MH Smolen JS Betteridge N Breedveld FC Burmester G Dorner T Ferraccioli G Gottenberg JE Isaacs J Kvien TK Mariette X Martin-Mola E Pavelka K Tak PP van der Heijde D van Vollenhoven RF Emery P Rituximab Consensus Expert Committee Updated consensus statement on the use of rituximab in patients with rheumatoid arthritis Ann Rheum Dis 2011 70 909 920 21378402 10.1136/ard.2010.144998 PMC3086093

[B65] Gottenberg JE Ravaud P Bardin T Cacoub P Cantagrel A Combe B Dougados M Flipo RM Godeau B Guillevin L Le Loet X Hachulla E Schaeverbeke T Sibilia J Baron G Mariette X AutoImmunity and Rituximab registry and French Society of Rheumatology Risk factors for severe infections in patients with rheumatoid arthritis treated with rituximab in the autoimmunity and rituximab registry Arthritis Rheum 2010 62 2625 2632 20506353 10.1002/art.27555

[B66] Fleischmann RM Progressive multifocal leukoencephalopathy following rituximab treatment in a patient with rheumatoid arthritis Arthritis Rheum 2009 60 3225 3228 19877057 10.1002/art.24906

[B67] Carson KR Evens AM Richey EA Habermann TM Focosi D Seymour JF Laubach J Bawn SD Gordon LI Winter JN Furman RR Vose JM Zelenetz AD Mamtani R Raisch DW Dorshimer GW Rosen ST Muro K Gottardi-Littell NR Talley RL Sartor O Green D Major EO Bennett CL Progressive multifocal leukoencephalopathy after rituximab therapy in HIV-negative patients: a report of 57 cases from the Research on Adverse Drug Events and Reports project Blood 2009 113 4834 4840 19264918 10.1182/blood-2008-10-186999 PMC2686134

[B68] Molloy ES Calabrese LH Progressive multifocal leukoencephalopathy associated with immunosuppressive therapy in rheumatic diseases: evolving role of biologic therapies Arthritis Rheum 2012 64 3043 3051 22422012 10.1002/art.34468

[B69] Linda H von Heijne A Major EO Ryschkewitsch C Berg J Olsson T Martin C Progressive multifocal leukoencephalopathy after natalizumab monotherapy N Engl J Med 2009 361 1081 1087 19741229 10.1056/NEJMoa0810316

[B70] Wenning W Haghikia A Laubenberger J Clifford DB Behrens PF Chan A Gold R Treatment of progressive multifocal leukoencephalopathy associated with natalizumab N Engl J Med 2009 361 1075 1080 19741228 10.1056/NEJMoa0810257

[B71] Eisenberg RA Jawad AF Boyer J Maurer K McDonald K Prak ET Sullivan KE Rituximab-treated patients have a poor response to influenza vaccination J Clin Immunol 2012 33 388 396 23064976 10.1007/s10875-012-9813-x PMC3565069

[B72] Yri OE Torfoss D Hungnes O Tierens A Waalen K Nordoy T Dudman S Kilander A Wader KF Ostenstad B Ekanger R Meyer P Kolstad A Rituximab blocks protective serologic response to influenza A (H1N1) 2009 vaccination in lymphoma patients during or within 6 months after treatment Blood 2011 118 6769 6771 22058114 10.1182/blood-2011-08-372649

[B73] Arad U Tzadok S Amir S Mandelboim M Mendelson E Wigler I Sarbagil-Maman H Paran D Caspi D Elkayam O The cellular immune response to influenza vaccination is preserved in rheumatoid arthritis patients treated with rituximab Vaccine 2011 29 1643 1648 21211590 10.1016/j.vaccine.2010.12.072

[B74] Looney RJ Anolik JH Campbell D Felgar RE Young F Arend LJ Sloand JA Rosenblatt J Sanz I B cell depletion as a novel treatment for systemic lupus erythematosus: a phase I/II dose-escalation trial of rituximab Arthritis Rheum 2004 50 2580 2589 15334472 10.1002/art.20430

[B75] Anolik JH Barnard J Cappione A Pugh-Bernard AE Felgar RE Looney RJ Sanz I Rituximab improves peripheral B cell abnormalities in human systemic lupus erythematosus Arthritis Rheum 2004 50 3580 3590 15529346 10.1002/art.20592

[B76] Leandro MJ Cambridge G Edwards JC Ehrenstein MR Isenberg DA B-cell depletion in the treatment of patients with systemic lupus erythematosus: a longitudinal analysis of 24 patients Rheumatology (Oxford) 2005 44 1542 1545 16188950 10.1093/rheumatology/kei080

[B77] Smith KG Jones RB Burns SM Jayne DR Long-term comparison of rituximab treatment for refractory systemic lupus erythematosus and vasculitis: Remission, relapse, and re-treatment Arthritis Rheum 2006 54 2970 2982 16947528 10.1002/art.22046

[B78] Cambridge G Leandro MJ Teodorescu M Manson J Rahman A Isenberg DA Edwards JC B cell depletion therapy in systemic lupus erythematosus: effect on autoantibody and antimicrobial antibody profiles Arthritis Rheum 2006 54 3612 3622 17075806 10.1002/art.22211

[B79] Albert D Dunham J Khan S Stansberry J Kolasinski S Tsai D Pullman-Mooar S Barnack F Striebich C Looney RJ Prak ET Kimberly R Zhang Y Eisenberg R Variability in the biological response to anti-CD20 B cell depletion in systemic lupus erythaematosus Ann Rheum Dis 2008 67 1724 1731 18250115 10.1136/ard.2007.083162

[B80] Lindholm C Borjesson-Asp K Zendjanchi K Sundqvist AC Tarkowski A Bokarewa M Longterm clinical and immunological effects of anti-CD20 treatment in patients with refractory systemic lupus erythematosus J Rheumatol 2008 35 826 833 18398943

[B81] Catapano F Chaudhry AN Jones RB Smith KG Jayne DW Long-term efficacy and safety of rituximab in refractory and relapsing systemic lupus erythematosus Nephrol Dial Transplant 2010 25 3586 3592 20466686 10.1093/ndt/gfq256

[B82] Terrier B Amoura Z Ravaud P Hachulla E Jouenne R Combe B Bonnet C Cacoub P Cantagrel A de Bandt M Fain O Fautrel B Gaudin P Godeau B Harle JR Hot A Kahn JE Lambotte O Larroche C Leone J Meyer O Pallot-Prades B Pertuiset E Quartier P Schaerverbeke T Sibilia J Somogyi A Soubrier M Vignon E Bader-Meunier B Safety and efficacy of rituximab in systemic lupus erythematosus: results from 136 patients from the French AutoImmunity and Rituximab registry Arthritis Rheum 2010 62 2458 2466 20506527 10.1002/art.27541

[B83] Jonsdottir T Gunnarsson I Mourao AF Lu TY van Vollenhoven RF Isenberg D Clinical improvements in proliferative vs membranous lupus nephritis following B-cell depletion: pooled data from two cohorts Rheumatology (Oxford) 2010 49 1502 1504 20427294 10.1093/rheumatology/keq055

[B84] Jonsdottir T Sundelin B Welin Henriksson E van Vollenhoven RF Gunnarsson I Rituximab-treated membranous lupus nephritis: clinical outcome and effects on electron dense deposits Ann Rheum Dis 2011 70 1172 1173 21367763 10.1136/ard.2010.129288

[B85] Moroni G Gallelli B Sinico RA Romano G Sinigaglia L Messa P Rituximab versus oral cyclophosphamide for treatment of relapses of proliferative lupus nephritis: a clinical observational study Ann Rheum Dis 2012 71 1751 1752 22586177 10.1136/annrheumdis-2012-201442

[B86] Diaz-Lagares C Croca S Sangle S Vital EM Catapano F Martinez-Berriotxoa A Garcia-Hernandez F Callejas-Rubio JL Rascon J D'Cruz D Jayne D Ruiz-Irastorza G Emery P Isenberg D Ramos-Casals M Khamashta MA UKBIOGEAS Registry Efficacy of rituximab in 164 patients with biopsy-proven lupus nephritis: pooled data from European cohorts Autoimmun Rev 2012 11 357 364 22032879 10.1016/j.autrev.2011.10.009

[B87] Merrill JT Neuwelt CM Wallace DJ Shanahan JC Latinis KM Oates JC Utset TO Gordon C Isenberg DA Hsieh HJ Zhang D Brunetta PG Efficacy and safety of rituximab in moderately-to-severely active systemic lupus erythematosus: the randomized, double-blind, phase II/III systemic lupus erythematosus evaluation of rituximab trial Arthritis Rheum 2010 62 222 233 20039413 10.1002/art.27233 PMC4548300

[B88] Merrill J Buyon J Furie R Latinis K Gordon C Hsieh HJ Brunetta P Assessment of flares in lupus patients enrolled in a phase II/III study of rituximab (EXPLORER) Lupus 2011 20 709 716 21478286 10.1177/0961203310395802

[B89] Rovin BH Furie R Latinis K Looney RJ Fervenza FC Sanchez-Guerrero J Maciuca R Zhang D Garg JP Brunetta P Appel G LUNAR Investigator Group Efficacy and safety of rituximab in patients with active proliferative lupus nephritis: the Lupus Nephritis Assessment with Rituximab study Arthritis Rheum 2012 64 1215 1226 22231479 10.1002/art.34359

[B90] Karnell JL Karnell FG Stephens GL Rajan B Morehouse C Li Y Swerdlow B Wilson M Goldbach-Mansky R Groves C Coyle AJ Herbst R Ettinger R Mycophenolic acid differentially impacts B cell function depending on the stage of differentiation J Immunol 2011 187 3603 3612 21873529 10.4049/jimmunol.1003319 PMC4180087

[B91] Herbst R Liu Z Jallal B Yao Y Biomarkers for systemic lupus erythematosus Int J Rheum Dis 2012 15 433 444 23083033 10.1111/j.1756-185X.2012.01764.x

[B92] Youinou P Devauchelle-Pensec V Pers JO Significance of B cells and B cell clonality in Sjogren's syndrome Arthritis Rheum 2010 62 2605 2610 20496425 10.1002/art.27564

[B93] Kallenberg CG Vissink A Kroese FG Abdulahad WH Bootsma H What have we learned from clinical trials in primary Sjogren's syndrome about pathogenesis? Arthritis Res Ther 2011 13 205 21371351 10.1186/ar3234 PMC3157640

[B94] Devauchelle-Pensec V Mariette X Jousse-Joulin S Berthelot J Perdriger A Hachulla E Puechal X Le Guern V Sibilia J Gottenberg J Chiche L Goeb V Hayem G Morel J Zarnitsky C Dubost J Pers J Nowak E Saraux A Tolerance and efficacy of rituximab in primary Sjogren syndrome (TEARS): results of a randomized controlled trial [abstract] Ann Rheum Dis 2012 71 Suppl 3 7521953334

[B95] Perez OA Patton T Novel therapies for pemphigus vulgaris: an overview Drugs Aging 2009 26 833 846 19761276 10.2165/11316810-000000000-00000

[B96] Delva E Tucker DK Kowalczyk AP The desmosome Cold Spring Harb Perspect Biol 2009 1 a002543 20066089 10.1101/cshperspect.a002543 PMC2742091

[B97] Mahoney MG Wang Z Rothenberger K Koch PJ Amagai M Stanley JR Explanations for the clinical and microscopic localization of lesions in pemphigus foliaceus and vulgaris J Clin Invest 1999 103 461 468 10021453 10.1172/JCI5252 PMC408100

[B98] Amagai M Stanley JR Desmoglein as a target in skin disease and beyond J Invest Dermatol 2012 132 3 Pt 2 776 784 22189787 10.1038/jid.2011.390 PMC3279627

[B99] Schmidt E Goebeler M Zillikens D Rituximab in severe pemphigus Ann N Y Acad Sci 2009 1173 683 691 19758216 10.1111/j.1749-6632.2009.04744.x

[B100] Mouquet H Musette P Gougeon ML Jacquot S Lemercier B Lim A Gilbert D Dutot I Roujeau JC D'Incan M Bedane C Tron F Joly P B-cell depletion immunotherapy in pemphigus: effects on cellular and humoral immune responses J Invest Dermatol 2008 128 2859 2869 18563177 10.1038/jid.2008.178

[B101] Gehrs BC Friedberg RC Autoimmune hemolytic anemia Am J Hematol 2002 69 258 271 11921020 10.1002/ajh.10062

[B102] McMillan R Antiplatelet antibodies in chronic immune thrombocytopenia and their role in platelet destruction and defective platelet production Hematol Oncol Clin North Am 2009 23 1163 1175 19932426 10.1016/j.hoc.2009.08.008

[B103] McCrae K Immune thrombocytopenia: no longer 'idiopathic' Cleve Clin J Med 2011 78 358 373 21632906 10.3949/ccjm.78gr.10005 PMC3410635

[B104] Garvey B Rituximab in the treatment of autoimmune haematological disorders Br J Haematol 2008 141 149 169 18318765 10.1111/j.1365-2141.2008.07054.x

[B105] Dierickx D Verhoef G Van Hoof A Mineur P Roest A Triffet A Kentos A Pierre P Boulet D Bries G Le PQ Janssens A Delannoy A Rituximab in autoimmune haemolytic anaemia and immune thrombocytopenic purpura: a Belgian retrospective multicentric study J Intern Med 2009 266 484 491 19549092 10.1111/j.1365-2796.2009.02126.x

[B106] Bussel JB Traditional and new approaches to the management of immune thrombocytopenia: issues of when and who to treat Hematol Oncol Clin North Am 2009 23 1329 1341 19932437 10.1016/j.hoc.2009.09.004

[B107] Auger S Duny Y Rossi JF Quittet P Rituximab before splenectomy in adults with primary idiopathic thrombocytopenic purpura: a meta-analysis Br J Haematol 2012 158 386 398 22612239 10.1111/j.1365-2141.2012.09169.x

[B108] Hasan A Michel M Patel V Stasi R Cunningham-Rundles S Leonard JP Bussel J Repeated courses of rituximab in chronic ITP: three different regimens Am J Hematol 2009 84 661 665 19731307 10.1002/ajh.21512 PMC2783818

[B109] Stasi R Stipa E Forte V Meo P Amadori S Variable patterns of response to rituximab treatment in adults with chronic idiopathic thrombocytopenic purpura Blood 2002 99 3872 3873 12014370 10.1182/blood-2002-02-0392

[B110] Kausar F Mustafa K Sweis G Sawaqed R Alawneh K Salloum R Badaracco M Niewold TB Sweiss NJ Ocrelizumab: a step forward in the evolution of B-cell therapy Expert Opin Biol Ther 2009 9 889 895 19463076 10.1517/14712590903018837

[B111] Genovese MC Kaine JL Lowenstein MB Del Giudice J Baldassare A Schechtman J Fudman E Kohen M Gujrathi S Trapp RG Sweiss NJ Spaniolo G Dummer W ACTION Study Group Ocrelizumab, a humanized anti-CD20 monoclonal antibody, in the treatment of patients with rheumatoid arthritis: a phase I/II randomized, blinded, placebo-controlled, doseranging study Arthritis Rheum 2008 58 2652 2661 18759293 10.1002/art.23732

[B112] Harigai M Tanaka Y Maisawa S JA21963 Study Group Safety and efficacy of various dosages of ocrelizumab in Japanese patients with rheumatoid arthritis with an inadequate response to methotrexate therapy: a placebo-controlled double-blind parallel-group study J Rheumatol 2012 39 486 495 22247354 10.3899/jrheum.110994

[B113] Rigby W Tony HP Oelke K Combe B Laster A von Muhlen CA Fisheleva E Martin C Travers H Dummer W Safety and efficacy of ocrelizumab in patients with rheumatoid arthritis and an inadequate response to methotrexate: results of a forty-eight-week randomized, double-blind, placebo-controlled, parallel-group phase III trial Arthritis Rheum 2012 64 350 359 21905001 10.1002/art.33317

[B114] Stohl W Gomez-Reino J Olech E Dudler J Fleischmann RM Zerbini CA Ashrafzadeh A Grzeschik S Bieraugel R Green J Francom S Dummer W Safety and efficacy of ocrelizumab in combination with methotrexate in MTX-naive subjects with rheumatoid arthritis: the phase III FILM trial Ann Rheum Dis 2012 71 1289 1296 22307942 10.1136/annrheumdis-2011-200706 PMC3396459

[B115] Tak PP Mease PJ Genovese MC Kremer J Haraoui B Tanaka Y Bingham CO Ashrafzadeh A Travers H Safa-Leathers S Kumar S Dummer W Safety and efficacy of ocrelizumab in patients with rheumatoid arthritis and an inadequate response to at least one tumor necrosis factor inhibitor: results of a forty-eight-week randomized, double-blind, placebo-controlled, parallel-group phase III trial Arthritis Rheum 2012 64 360 370 22389919 10.1002/art.33353

[B116] Lopez-Diego RS Weiner HL Novel therapeutic strategies for multiple sclerosis--a multifaceted adversary Nat Rev Drug Discov 2008 7 909 925 18974749 10.1038/nrd2358

[B117] von Budingen HC Bar-Or A Zamvil SS B cells in multiple sclerosis: connecting the dots Curr Opin Immunol 2011 23 713 720 21983151 10.1016/j.coi.2011.09.003 PMC4188435

[B118] Lassmann H Bruck W Lucchinetti CF The immunopathology of multiple sclerosis: an overview Brain Pathol 2007 17 210 218 17388952 10.1111/j.1750-3639.2007.00064.x PMC8095582

[B119] Barun B Bar-Or A Treatment of multiple sclerosis with anti-CD20 antibodies Clin Immunol 2012 142 31 37 21555250 10.1016/j.clim.2011.04.005

[B120] Bartok B Silverman GJ Development of anti-CD20 therapy for multiple sclerosis Exp Cell Res 2011 317 1312 1318 21510932 10.1016/j.yexcr.2011.04.002 PMC3266104

[B121] . Gensicke H Leppert D Yaldizli O Lindberg RL Mehling M Kappos L Kuhle J Monoclonal antibodies and recombinant immunoglobulins for the treatment of multiple sclerosis CNS Drugs 2012 26 11 37 22171583 10.2165/11596920-000000000-00000

[B122] Hauser SL Waubant E Arnold DL Vollmer T Antel J Fox RJ Bar-Or A Panzara M Sarkar N Agarwal S Langer-Gould A Smith CH HERMES Trial Group B-cell depletion with rituximab in relapsing-remitting multiple sclerosis N Engl J Med 2008 358 676 688 18272891 10.1056/NEJMoa0706383

[B123] Hawker K O'Connor P Freedman MS Calabresi PA Antel J Simon J Hauser S Waubant E Vollmer T Panitch H Zhang J Chin P Smith CH OLYMPUS trial group Rituximab in patients with primary progressive multiple sclerosis: results of a randomized double-blind placebo-controlled multicenter trial Ann Neurol 2009 66 460 471 19847908 10.1002/ana.21867

[B124] Kappos L Li D Calabresi PA O'Connor P Bar-Or A Barkhof F Yin M Leppert D Glanzman R Tinbergen J Hauser SL Ocrelizumab in relapsing-remitting multiple sclerosis: a phase 2, randomised, placebo-controlled, multicentre trial Lancet 2011 378 1779 1787 22047971 10.1016/S0140-6736(11)61649-8

[B125] Castillo J Milani C Mendez-Allwood D Ofatumumab, a second-generation anti-CD20 monoclonal antibody, for the treatment of lymphoproliferative and autoimmune disorders Expert Opin Investig Drugs 2009 18 491 500 10.1517/13543780902832679 19335277

[B126] Teeling JL Mackus WJ Wiegman LJ van den Brakel JH Beers SA French RR van Meerten T Ebeling S Vink T Slootstra JW Parren PW Glennie MJ van de Winkel JG The biological activity of human CD20 monoclonal antibodies is linked to unique epitopes on CD20 J Immunol 2006 177 362 371 16785532 10.4049/jimmunol.177.1.362

[B127] Teeling JL French RR Cragg MS van den Brakel J Pluyter M Huang H Chan C Parren PW Hack CE Dechant M Valerius T van de Winkel JG Glennie MJ Characterization of new human CD20 monoclonal antibodies with potent cytolytic activity against non-Hodgkin lymphomas Blood 2004 104 1793 1800 15172969 10.1182/blood-2004-01-0039

[B128] Pawluczkowycz AW Beurskens FJ Beum PV Lindorfer MA van de Winkel JG Parren PW Taylor RP Binding of submaximal C1q promotes complement-dependent cytotoxicity (CDC) of B cells opsonized with anti-CD20 mAbs ofatumumab (OFA) or rituximab (RTX): considerably higher levels of CDC are induced by OFA than by RTX J Immunol 2009 183 749 758 19535640 10.4049/jimmunol.0900632

[B129] Ostergaard M Baslund B Rigby W Rojkovich B Jorgensen C Dawes PT Wiell C Wallace DJ Tamer SC Kastberg H Petersen J Sierakowski S Ofatumumab, a human anti-CD20 monoclonal antibody, for treatment of rheumatoid arthritis with an inadequate response to one or more disease-modifying antirheumatic drugs: results of a randomized, double-blind, placebo-controlled, phase I/II study Arthritis Rheum 2010 62 2227 2238 20506254 10.1002/art.27524

[B130] Taylor PC Quattrocchi E Mallett S Kurrasch R Petersen J Chang DJ Ofatumumab, a fully human anti-CD20 monoclonal antibody, in biological-naive, rheumatoid arthritis patients with an inadequate response to methotrexate: a randomised, double-blind, placebocontrolled clinical trial Ann Rheum Dis 2011 70 2119 2125 21859685 10.1136/ard.2011.151522 PMC3212699

[B131] Sorensen PS Drulovic J Havrdova E Lisby S Graff O Shackelford S Magnetic resonance imaging (MRI) efficacy of ofatumumab in relapsing-remitting multiple sclerosis (RRMS) - 24-week results of a phase II study [abstract] Presented at 26th Congress of the European Committee for Treatment and Research in Multiple Sclerosis (ECTRIMS) & 15th Annual Conference of Rehabilitation in MS (RIMS) 2010 Gothenburg, Sweden October 13-16

[B132] Tedder TF CD19: a promising B cell target for rheumatoid arthritis Nat Rev Rheumatol 2009 5 572 577 19798033 10.1038/nrrheum.2009.184

[B133] Engel P Zhou LJ Ord DC Sato S Koller B Tedder TF Abnormal B lymphocyte development, activation, and differentiation in mice that lack or overexpress the CD19 signal transduction molecule Immunity 1995 3 39 50 7542548 10.1016/1074-7613(95)90157-4

[B134] Sato S Steeber DA Tedder TF The CD19 signal transduction molecule is a response regulator of B-lymphocyte differentiation Proc Natl Acad Sci USA 1995 92 11558 11562 8524803 10.1073/pnas.92.25.11558 PMC40441

[B135] Yazawa N Hamaguchi Y Poe JC Tedder TF Immunotherapy using unconjugated CD19 monoclonal antibodies in animal models for B lymphocyte malignancies and autoimmune disease Proc Natl Acad Sci USA 2005 102 15178 15183 16217038 10.1073/pnas.0505539102 PMC1257712

[B136] Tedder TF Poe JC Haas KM CD22: a multifunctional receptor that regulates B lymphocyte survival and signal transduction Adv Immunol 2005 88 1 50 16227086 10.1016/S0065-2776(05)88001-0

[B137] Walker JA Smith KG CD22: an inhibitory enigma Immunology 2008 123 314 325 18067554 10.1111/j.1365-2567.2007.02752.x PMC2433339

[B138] Han K Kim Y Lee J Lim J Lee KY Kang CS Kim WI Kim BK Shim SI Kim SM Human basophils express CD22 without expression of CD19 Cytometry 1999 37 178 183 10520197 10.1002/(sici)1097-0320(19991101)37:3<178::aid-cyto3>3.3.co;2-q

[B139] Reineks EZ Osei ES Rosenberg A Auletta J Meyerson HJ CD22 expression on blastic plasmacytoid dendritic cell neoplasms and reactivity of anti- CD22 antibodies to peripheral blood dendritic cells Cytometry B Clin Cytom 2009 76 237 248 19382197 10.1002/cyto.b.20469

[B140] Acon-Laws M Bayerl MG Ehman C Malysz J Boyer C Basophils and plasmacytoid dendritic cells are potential sources for error in flow cytometric monitoring of patients receiving anti-CD22 therapies. AKA not all anti-CD22 antibodies are created equal Am J Hematol 2011 86 891 892 21922531 10.1002/ajh.22130

[B141] Leung SO Goldenberg DM Dion AS Pellegrini MC Shevitz J Shih LB Hansen HJ Construction and characterization of a humanized, internalizing, B-cell (CD22)-specific, leukemia/lymphoma antibody, LL2 Mol Immunol 1995 32 1413 1427 8643111 10.1016/0161-5890(95)00080-1

[B142] Carnahan J Stein R Qu Z Hess K Cesano A Hansen HJ Goldenberg DM Epratuzumab, a CD22-targeting recombinant humanized antibody with a different mode of action from rituximab Mol Immunol 2007 44 1331 1341 16814387 10.1016/j.molimm.2006.05.007

[B143] Carnahan J Wang P Kendall R Chen C Hu S Boone T Juan T Talvenheimo J Montestruque S Sun J Elliott G Thomas J Ferbas J Kern B Briddell R Leonard JP Cesano A Epratuzumab, a humanized monoclonal antibody targeting CD22: characterization of in vitro properties Clin Cancer Res 2003 9 10 Pt 2 3982S 3990S 14506197

[B144] Steinfeld SD Tant L Burmester GR Teoh NK Wegener WA Goldenberg DM Pradier O Epratuzumab (humanised anti-CD22 antibody) in primary Sjogren's syndrome: an open-label phase I/II study Arthritis Res Ther 2006 8 R129 16859536 10.1186/ar2018 PMC1779377

[B145] Dorner T Kaufmann J Wegener WA Teoh N Goldenberg DM Burmester GR Initial clinical trial of epratuzumab (humanized anti-CD22 antibody) for immunotherapy of systemic lupus erythematosus Arthritis Res Ther 2006 8 R74 16630358 10.1186/ar1942 PMC1526638

[B146] Wallace D Hobbs K Houssiau F Strand V Tak P Wegener W Kelley L Barry A Randomized controlled trials of epratuzumab (anti-CD22 mAb targeting B-cells) reveal clinically meaningful reductions in corticosteroid (CS) use with favorable safety profile in moderate and severe flaring SLE patients [abstract] Ann Rheum Dis 2008 67 Suppl 2 21217526555

[B147] Strand V Gordon C Kalunian K Coteur G Barry A Keininger D Wegener W Petri M Randomized controlled trials (RCTs) of epratuzumab (anti-CD22 mAb targeting B-cells) show meaningful improvements in health related-quality of life (HRQOL) in SLE patients (pts) with high disease activity and low baseline (BL) self-report measures [abstract] Ann Rheum Dis 2008 67 Suppl 2 21217526555

[B148] Petri M Hobbs K Gordon C Strand V Wallace D Kelley L Wegener W Barry A Randomized controlled trials (RCTs) of epratuzumab (anti-CD22 mAb targeting B-cells) reveal clinically meaningful improvements in patients (pts) with moderate/severe SLE flares [abstract] Ann Rheum Dis 2008 67 Suppl 2 53

[B149] Wallace DJ Kalunian KC Petri MA Strand V Kilgallen B Kelley L Gordon CP Epratuzumab demonstrates clinically meaningful improvements in patients with moderate to severe systemic lupus erythematosus (SLE): results from EMBLEM™, a phase IIb study [abstract] Ann Rheum Dis 2010 69 Suppl 3 559

[B150] Sims GP Ettinger R Shirota Y Yarboro CH Illei GG Lipsky PE Identification and characterization of circulating human transitional B cells Blood 2005 105 4390 4398 15701725 10.1182/blood-2004-11-4284 PMC1895038

[B151] Suryani S Fulcher DA Santner-Nanan B Nanan R Wong M Shaw PJ Gibson J Williams A Tangye SG Differential expression of CD21 identifies developmentally and functionally distinct subsets of human transitional B cells Blood 2010 115 519 529 19965666 10.1182/blood-2009-07-234799

[B152] Jacobi AM Huang W Wang T Freimuth W Sanz I Furie R Mackay M Aranow C Diamond B Davidson A Effect of long-term belimumab treatment on B cells in systemic lupus erythematosus: extension of a phase II, doubleblind, placebo-controlled, dose-ranging study Arthritis Rheum 2010 62 201 210 20039404 10.1002/art.27189 PMC2857977

[B153] He B Xu W Santini PA Polydorides AD Chiu A Estrella J Shan M Chadburn A Villanacci V Plebani A Knowles DM Rescigno M Cerutti A Intestinal bacteria trigger T cell-independent immunoglobulin A(2) class switching by inducing epithelial-cell secretion of the cytokine APRIL Immunity 2007 26 812 826 17570691 10.1016/j.immuni.2007.04.014

[B154] Tangye SG Liu YJ Aversa G Phillips JH de Vries JE Identification of functional human splenic memory B cells by expression of CD148 and CD27 J Exp Med 1998 188 1691 1703 9802981 10.1084/jem.188.9.1691 PMC2212517

[B155] Jego G Bataille R Pellat-Deceunynck C Interleukin-6 is a growth factor for nonmalignant human plasmablasts Blood 2001 97 1817 1822 11238125 10.1182/blood.v97.6.1817

[B156] Kuchen S Robbins R Sims GP Sheng C Phillips TM Lipsky PE Ettinger R Essential role of IL-21 in B cell activation, expansion, and plasma cell generation during CD4^+ ^T cell-B cell collaboration J Immunol 2007 179 5886 5896 17947662 10.4049/jimmunol.179.9.5886

[B157] Avery DT Deenick EK Ma CS Suryani S Simpson N Chew GY Chan TD Palendira U Bustamante J Boisson-Dupuis S Choo S Bleasel KE Peake J King C French MA Engelhard D Al-Hajjar S Al-Muhsen S Magdorf K Roesler J Arkwright PD Hissaria P Riminton DS Wong M Brink R Fulcher DA Casanova JL Cook MC Tangye SG B cell-intrinsic signaling through IL-21 receptor and STAT3 is required for establishing long-lived antibody responses in humans J Exp Med 2010 207 155 171 20048285 10.1084/jem.20091706 PMC2812540

[B158] Bryant VL Ma CS Avery DT Li Y Good KL Corcoran LM de Waal Malefyt R Tangye SG Cytokine-mediated regulation of human B cell differentiation into Ig-secreting cells: predominant role of IL-21 produced by CXCR5^+ ^T follicular helper cells J Immunol 2007 179 8180 8190 18056361 10.4049/jimmunol.179.12.8180

[B159] Ettinger R Sims GP Fairhurst AM Robbins R da Silva YS Spolski R Leonard WJ Lipsky PE IL-21 induces differentiation of human naive and memory B cells into antibody-secreting plasma cells J Immunol 2005 175 7867 7879 16339522 10.4049/jimmunol.175.12.7867

[B160] Ettinger R Kuchen S Lipsky PE The role of IL-21 in regulating B-cell function in health and disease Immunol Rev 2008 223 60 86 18613830 10.1111/j.1600-065X.2008.00631.x

[B161] Yoon SO Zhang X Berner P Choi YS IL-21 and IL-10 have redundant roles but differential capacities at different stages of plasma cell generation from human germinal center B cells J Leukoc Biol 2009 86 1311 1318 19762555 10.1189/jlb.0409268

[B162] Ettinger R Sims GP Robbins R Withers D Fischer RT Grammer AC Kuchen S Lipsky PE IL-21 and BAFF/BLyS synergize in stimulating plasma cell differentiation from a unique population of human splenic memory B cells J Immunol 2007 178 2872 2882 17312131 10.4049/jimmunol.178.5.2872

[B163] Doreau A Belot A Bastid J Riche B Trescol-Biemont MC Ranchin B Fabien N Cochat P Pouteil-Noble C Trolliet P Durieu I Tebib J Kassai B Ansieau S Puisieux A Eliaou JF Bonnefoy-Berard N Interleukin 17 acts in synergy with B cell-activating factor to influence B cell biology and the pathophysiology of systemic lupus erythematosus Nat Immunol 2009 10 778 785 19483719 10.1038/ni.1741

[B164] Avery DT Kalled SL Ellyard JI Ambrose C Bixler SA Thien M Brink R Mackay F Hodgkin PD Tangye SG BAFF selectively enhances the survival of plasmablasts generated from human memory B cells J Clin Invest 2003 112 286 297 12865416 10.1172/JCI18025 PMC164292

[B165] Zacks Equity Research CHMP Backs Benlysta 2011 Zacks Investment Research http://www.zacks.com

[B166] Baker KP Edwards BM Main SH Choi GH Wager RE Halpern WG Lappin PB Riccobene T Abramian D Sekut L Sturm B Poortman C Minter RR Dobson CL Williams E Carmen S Smith R Roschke V Hilbert DM Vaughan TJ Albert VR Generation and characterization of LymphoStat-B, a human monoclonal antibody that antagonizes the bioactivities of B lymphocyte stimulator Arthritis Rheum 2003 48 3253 3265 14613291 10.1002/art.11299

[B167] Furie R Petri M Zamani O Cervera R Wallace DJ Tegzova D Sanchez-Guerrero J Schwarting A Merrill JT Chatham WW Stohl W Ginzler EM Hough DR Zhong ZJ Freimuth W van Vollenhoven RF BLISS-76 Study Group A phase III, randomized, placebo-controlled study of belimumab, a monoclonal antibody that inhibits B lymphocyte stimulator, in patients with systemic lupus erythematosus Arthritis Rheum 2011 63 3918 3930 22127708 10.1002/art.30613 PMC5007058

[B168] Navarra SV Guzman RM Gallacher AE Hall S Levy RA Jimenez RE Li EK Thomas M Kim HY Leon MG Tanasescu C Nasonov E Lan JL Pineda L Zhong ZJ Freimuth W Petri MA BLISS-52 Study Group Efficacy and safety of belimumab in patients with active systemic lupus erythematosus: a randomised, placebo-controlled, phase 3 trial Lancet 2011 377 721 731 21296403 10.1016/S0140-6736(10)61354-2

[B169] Manzi S Sanchez-Guerrero J Merrill JT Furie R Gladman D Navarra SV Ginzler EM D'Cruz DP Doria A Cooper S Zhong ZJ Hough D Freimuth W Petri MA on behalf of the BLISS-52 and BLISS-76 Study Groups Effects of belimumab, a B lymphocyte stimulator-specific inhibitor, on disease activity across multiple organ domains in patients with systemic lupus erythematosus: combined results from two phase III trials Ann Rheum Dis 2012 71 1833 1838 22550315 10.1136/annrheumdis-2011-200831 PMC3465857

[B170] Boyce EG Fusco BE Belimumab: review of use in systemic lupus erythematosus Clin Ther 2012 34 1006 1022 22464040 10.1016/j.clinthera.2012.02.028

[B171] Davidson A Targeting BAFF in autoimmunity Curr Opin Immunol 2010 22 732 739 20970975 10.1016/j.coi.2010.09.010 PMC2997938

[B172] Gross JA Dillon SR Mudri S Johnston J Littau A Roque R Rixon M Schou O Foley KP Haugen H McMillen S Waggie K Schreckhise RW Shoemaker K Vu T Moore M Grossman A Clegg CH TACI-Ig neutralizes molecules critical for B cell development and autoimmune disease. impaired B cell maturation in mice lacking BLyS Immunity 2001 15 289 302 11520463 10.1016/s1074-7613(01)00183-2

[B173] Dall'Era M Chakravarty E Wallace D Genovese M Weisman M Kavanaugh A Kalunian K Dhar P Vincent E Pena-Rossi C Wofsy D Reduced B lymphocyte and immunoglobulin levels after atacicept treatment in patients with systemic lupus erythematosus: results of a multicenter, phase Ib, doubleblind, placebo-controlled, dose-escalating trial Arthritis Rheum 2007 56 4142 4150 18050206 10.1002/art.23047

[B174] Pena-Rossi C Nasonov E Stanislav M Yakusevich V Ershova O Lomareva N Saunders H Hill J Nestorov I An exploratory dose-escalating study investigating the safety, tolerability, pharmacokinetics and pharmacodynamics of intravenous atacicept in patients with systemic lupus erythematosus Lupus 2009 18 547 555 19395457 10.1177/0961203309102803 PMC3835146

[B175] Nestorov I Papasouliotis O Pena Rossi C Munafo A Pharmacokinetics and immunoglobulin response of subcutaneous and intravenous atacicept in patients with systemic lupus erythematosus J Pharm Sci 2010 99 524 538 19743503 10.1002/jps.21839

[B176] Genovese MC Kinnman N de La Bourdonnaye G Pena Rossi C Tak PP Atacicept in patients with rheumatoid arthritis and an inadequate response to tumor necrosis factor antagonist therapy: results of a phase II, randomized, placebo-controlled, dose-finding trial Arthritis Rheum 2011 63 1793 1803 21452293 10.1002/art.30373

[B177] van Vollenhoven RF Kinnman N Vincent E Wax S Bathon J Atacicept in patients with rheumatoid arthritis and an inadequate response to methotrexate: results of a phase II, randomized, placebo-controlled trial Arthritis Rheum 2011 63 1782 1792 21452294 10.1002/art.30372

[B178] Fernandez L Salinas GF Rocha C Carvalho-Pinto CE Yeremenko N Papon L Medema JP Combe B Morel J Baeten D Hahne M The TNF family member APRIL dampens collagen-induced arthritis Ann Rheum Dis 2012 in press 10.1136/annrheumdis-2012-202382 23178293

[B179] Spolski R Leonard WJ Interleukin-21: basic biology and implications for cancer and autoimmunity Annu Rev Immunol 2008 26 57 79 17953510 10.1146/annurev.immunol.26.021607.090316

[B180] Jones JL Phuah CL Cox AL Thompson SA Ban M Shawcross J Walton A Sawcer SJ Compston A Coles AJ IL-21 drives secondary autoimmunity in patients with multiple sclerosis, following therapeutic lymphocyte depletion with alemtuzumab (Campath-1H) J Clin Invest 2009 119 2052 2061 19546505 10.1172/JCI37878 PMC2701868

[B181] Caruso R Botti E Sarra M Esposito M Diluvio L Giustizieri ML Pacciani V Mazzotta A Campione E Macdonald TT Chimenti S Pallone F Costanzo A Monteleone G Involvement of interleukin-21 in the epidermal hyperplasia of psoriasis Nat Med 2009 15 1013 1015 19684581 10.1038/nm.1995

[B182] Rasmussen TK Andersen T Hvid M Hetland ML Horslev-Petersen K Stengaard-Pedersen K Holm CK Deleuran B Increased interleukin 21 (IL-21) and IL-23 are associated with increased disease activity and with radiographic status in patients with early rheumatoid arthritis J Rheumatol 2010 37 2014 2020 20682664 10.3899/jrheum.100259

[B183] Kang KY Kim HO Kwok SK Ju JH Park KS Sun DI Jhun JY Oh HJ Park SH Kim HY Impact of interleukin-21 in the pathogenesis of primary Sjogren's syndrome: increased serum levels of interleukin-21 and its expression in the labial salivary glands Arthritis Res Ther 2011 13 R179 22030011 10.1186/ar3504 PMC3308114

[B184] Tzartos JS Craner MJ Friese MA Jakobsen KB Newcombe J Esiri MM Fugger L IL-21 and IL-21 receptor expression in lymphocytes and neurons in multiple sclerosis brain Am J Pathol 2011 178 794 802 21281812 10.1016/j.ajpath.2010.10.043 PMC3032888

[B185] Dolff S Abdulahad WH Westra J Doornbos-van der Meer B Limburg PC Kallenberg CG Bijl M Increase in IL-21 producing T-cells in patients with systemic lupus erythematosus Arthritis Res Ther 2011 13 R157 21959034 10.1186/ar3474 PMC3308088

[B186] Gottenberg JE Dayer JM Lukas C Ducot B Chiocchia G Cantagrel A Saraux A Roux-Lombard P Mariette X Serum IL-6 and IL-21 are associated with markers of B cell activation and structural progression in early rheumatoid arthritis: results from the ESPOIR cohort Ann Rheum Dis 2012 71 1243 1248 22532637 10.1136/annrheumdis-2011-200975

[B187] Maurer MF Garrigues U Jaspers SR Meengs B Rixon MW Stevens BL Lewis KB Julien SH Bukowski TR Wolf AC Hamacher NB Snavely M Dillon SR Generation and characterization of human anti-human IL-21 neutralizing monoclonal antibodies MAbs 2012 4 69 83 22327431 10.4161/mabs.4.1.18713 PMC3338942

[B188] Chittasupho C Siahaan TJ Vines CM Berkland C Autoimmune therapies targeting costimulation and emerging trends in multivalent therapeutics Ther Deliv 2011 2 873 889 21984960 10.4155/tde.11.60 PMC3186944

[B189] Grimbacher B Hutloff A Schlesier M Glocker E Warnatz K Drager R Eibel H Fischer B Schaffer AA Mages HW Kroczek RA Peter HH Homozygous loss of ICOS is associated with adult-onset common variable immunodeficiency Nat Immunol 2003 4 261 268 12577056 10.1038/ni902

[B190] Dong C Temann UA Flavell RA Cutting edge: critical role of inducible costimulator in germinal center reactions J Immunol 2001 166 3659 3662 11238604 10.4049/jimmunol.166.6.3659

[B191] Law C Grewal I Grewal I Therapeutic interventions targeting CD40L (CD154) and CD40: the opportunities and challenges Therapeutic Targets of the TNF Superfamily (Advances in Experimental Medicine and Biology Volume 647) 2009 8 New York, NY: Landes Bioscience and Springer Science+Business Media 36 10.1007/978-0-387-89520-8_2 19760064

[B192] Aruffo A Farrington M Hollenbaugh D Li X Milatovich A Nonoyama S Bajorath J Grosmaire LS Stenkamp R Neubauer M The CD40 ligand, gp39, is defective in activated T cells from patients with X-linked hyper-IgM syndrome Cell 1993 72 291 300 7678782 10.1016/0092-8674(93)90668-g

[B193] Farrington M Grosmaire LS Nonoyama S Fischer SH Hollenbaugh D Ledbetter JA Noelle RJ Aruffo A Ochs HD CD40 ligand expression is defective in a subset of patients with common variable immunodeficiency Proc Natl Acad Sci USA 1994 91 1099 1103 7508119 10.1073/pnas.91.3.1099 PMC521461

[B194] Alaaeddine N Hassan GS Yacoub D Mourad W CD154: an immunoinflammatory mediator in systemic lupus erythematosus and rheumatoid arthritis Clin Dev Immunol 2012 2012 490148 22110533 10.1155/2012/490148 PMC3202102

[B195] Sullivan BA Green CL Zhang M Abbott C Belouski S Thomas G Gorski K A flow cytometric receptor occupancy assay demonstrates dose-dependent blockade of B7RP-1 by AMG 557 on circulating B cells from SLE subjects [abstract] Arthritis Rheum 2010 62 Suppl 10 1141

[B196] Hu YL Metz DP Chung J Siu G Zhang M B7RP-1 blockade ameliorates autoimmunity through regulation of follicular helper T cells J Immunol 2009 182 1421 1428 19155489 10.4049/jimmunol.182.3.1421

[B197] Kawamoto M Harigai M Hara M Kawaguchi Y Tezuka K Tanaka M Sugiura T Katsumata Y Fukasawa C Ichida H Higami S Kamatani N Expression and function of inducible co-stimulator in patients with systemic lupus erythematosus: possible involvement in excessive interferon-gamma and anti-double-stranded DNA antibody production Arthritis Res Ther 2006 8 R62 16563187 10.1186/ar1928 PMC1526621

[B198] Carlesso G Taylor D Herbst R Targeting inducible T-cell co-stimulator in autoimmune diseases - new evidence supporting its critical function in the maintenance of secondary lymphoid tissue architecture Eur Musculoskeletal Rev 2011 6 248 252

[B199] Robles-Carrillo L Meyer T Hatfield M Desai H Davila M Langer F Amaya M Garber E Francis JL Hsu YM Amirkhosravi A Anti-CD40L immune complexes potently activate platelets in vitro and cause thrombosis in FCGR2A transgenic mice J Immunol 2010 185 1577 1583 20585032 10.4049/jimmunol.0903888

[B200] Wakefield I Peters C Burkly L Garber E Ferrant J Taylor F Su L CDP7657, a monovalent Fab PEG anti-CD40L antibody, inhibits immune responses in both HuSCID mice and non-human primates. [abstract] Arthritis Rheum 2010 62 Suppl 10 1245

[B201] Sanz I Lee FE B cells as therapeutic targets in SLE Nat Rev Rheumatol 2010 6 326 337 20520647 10.1038/nrrheum.2010.68 PMC3934759

[B202] Huang H Benoist C Mathis D Rituximab specifically depletes short-lived autoreactive plasma cells in a mouse model of inflammatory arthritis Proc Natl Acad Sci USA 2010 107 4658 4663 20176942 10.1073/pnas.1001074107 PMC2842072

[B203] Hiepe F Dorner T Hauser AE Hoyer BF Mei H Radbruch A Long-lived autoreactive plasma cells drive persistent autoimmune inflammation Nat Rev Rheumatol 2011 7 170 178 21283146 10.1038/nrrheum.2011.1

[B204] Owczarczyk K Lal P Abbas AR Wolslegel K Holweg CT Dummer W Kelman A Brunetta P Lewin-Koh N Sorani M Leong D Fielder P Yocum D Ho C Ortmann W Townsend MJ Behrens TW A plasmablast biomarker for nonresponse to antibody therapy to CD20 in rheumatoid arthritis Sci Transl Med 2011 3 101ra92 10.1126/scitranslmed.3002432 21937757

[B205] Neubert K Meister S Moser K Weisel F Maseda D Amann K Wiethe C Winkler TH Kalden JR Manz RA Voll RE The proteasome inhibitor bortezomib depletes plasma cells and protects mice with lupus-like disease from nephritis Nat Med 2008 14 748 755 18542049 10.1038/nm1763

[B206] Voll RE Alexander T Peukert R Rubbert A Rech J Braun T Wiesener M Eckardt K-U Hoyer B Taddeo A Reisch A Burmester G-R Radbruch A Schett G Hiepe F Successful treatment of refractory SLE patients with the proteasome inhibitor Bortezomib - a case series [abstract] Presented at the European League Against Rheumatism (EULAR) Conference 2012 Berlin, Germany June 6-9

[B207] Cross AH Stark JL Lauber J Ramsbottom MJ Lyons JA Rituximab reduces B cells and T cells in cerebrospinal fluid of multiple sclerosis patients J Neuroimmunol 2006 180 63 70 16904756 10.1016/j.jneuroim.2006.06.029 PMC1769354

[B208] Piccio L Naismith RT Trinkaus K Klein RS Parks BJ Lyons JA Cross AH Changes in B- and T-lymphocyte and chemokine levels with rituximab treatment in multiple sclerosis Arch Neurol 2010 67 707 714 20558389 10.1001/archneurol.2010.99 PMC2918395

[B209] van de Veerdonk FL Lauwerys B Marijnissen RJ Timmermans K Di Padova F Koenders MI Gutierrez-Roelens I Durez P Netea MG van der Meer JW van den Berg WB Joosten LA The anti-CD20 antibody rituximab reduces the Th17 cell response Arthritis Rheum 2011 63 1507 1516 21400475 10.1002/art.30314

